# Benchmarking Spatial Clustering Methods for Mass Spectrometry-Based Spatial Metabolomics

**DOI:** 10.3390/metabo16050348

**Published:** 2026-05-21

**Authors:** Yunning Lu, Zhanlong Mei, Haoke Deng, Yun Zhao, Chunlu Feng, Siqi Liu

**Affiliations:** 1 School of Biology and Biological Engineering, South China University of Technology, Guangzhou 510006, China; 202320148479@mail.scut.edu.cn; 2 BGI Genomics, Shenzhen 518083, China; meizhanlong@genomics.cn (Z.M.); denghaoke@genomics.cn (H.D.); zhaoyun@genomics.cn (Y.Z.); fengchunlu@genomics.cn (C.F.)

**Keywords:** spatial metabolomics, mass spectrometry imaging, clustering benchmarking, spatial continuity, inter-cluster heterogeneity, online platform

## Abstract

**Background:** Mass spectrometry imaging (MSI) enables in situ mapping of metabolite distributions within tissues, and spatial clustering is a key step for delineating metabolically distinct regions. Nevertheless, spatial clustering methods have not been systematically benchmarked for spatial metabolomics data. **Methods**: Here, we evaluated the effects of ion filtering and clustering method selection on clustering performance and established a dual-metric framework that jointly assesses the spatial continuity of cluster labels and inter-cluster metabolic heterogeneity. We benchmarked 30 clustering algorithms across 12 heterogeneous MSI datasets spanning three major ion sources, four mass analyzers, and multiple spatial resolutions, covering approaches from non-spatial methods to advanced spatially aware models. **Results**: Noise filtering markedly improved the spatial continuity of results generated by non-spatial methods (mean improvement, approximately 28%) but provided limited benefit for spatially aware methods. Across the 12 datasets, a median of only 11 methods satisfied both evaluation criteria simultaneously, whereas SSC and DRSC met the dual-metric thresholds in at least nine datasets. In the mbrain2_pos50 dataset, the top-ranked method based on the composite dual-metric score achieved 22% higher concordance between cluster assignments and cell-type annotations than the lowest-ranked method. **Conclusions**: Together, the proposed evaluation framework and the online platform SMcluster provide a standardized resource for benchmarking and selecting MSI clustering methods. Our results highlight the critical roles of preprocessing and method selection in determining spatial clustering performance and offer practical guidance for spatial metabolomics studies.

## 1. Introduction

Spatial metabolomics offers a unique molecular perspective for elucidating disease mechanisms and cellular heterogeneity by mapping the in situ distribution of metabolites within the tissue microenvironment [1,2,3]. Major mass spectrometry imaging (MSI) platforms include matrix-assisted laser desorption/ionization mass spectrometry imaging (MALDI-MSI) [4], desorption electrospray ionization mass spectrometry imaging (DESI-MSI), and air flow-assisted desorption electrospray ionization mass spectrometry imaging (AFADESI-MSI) [5]. These platforms differ markedly in spatial resolution, detection sensitivity, and sample compatibility. In addition, instrument-specific noise characteristics—such as matrix effects in MALDI and ion suppression in DESI—together with the high dimensionality of MSI data, which often comprise thousands of ions, pose substantial challenges for downstream analysis. This complexity highlights the need for computational frameworks tailored to spatial metabolomics.

Within the MSI analysis workflow, spatial clustering is a fundamental step for identifying tissue regions with distinct metabolic characteristics, thereby supporting downstream differential analysis, regional functional annotation, and biomarker discovery [6,7,8]. A broad range of clustering strategies has been proposed, including early approaches that combine dimensionality reduction with conventional clustering [9], spatially aware methods that incorporate neighborhood information [10,11], and more recent graph neural network models that capture complex spatial dependencies [12]. At the same time, advanced methods originally developed for spatial transcriptomics are increasingly being adapted for MSI analysis [13,14]. Despite this rapid methodological expansion, the suitability of different clustering methods for diverse spatial metabolomics datasets remains unclear. Although several benchmarking studies have been conducted in spatial transcriptomics [15,16,17,18], the substantial differences between transcriptomic and metabolomic measurements limit the direct transferability of those conclusions to spatial metabolomics. Moreover, most existing MSI clustering studies focus on introducing new methods and typically compare only 2–5 baseline methods [10,11,12,14,19,20]; consequently, no study has yet systematically benchmarked 30 clustering methods across more than 10 heterogeneous MSI datasets. In addition, the effects of preprocessing strategies, particularly noise filtering, on clustering performance remain insufficiently quantified [21,22,23], and the absence of a unified evaluation framework hinders cross-study comparison.

To address these gaps, we systematically evaluated 30 clustering methods across 12 MSI datasets spanning three ion sources, multiple spatial resolutions, and diverse tissue types. We established a dual-metric evaluation framework that jointly measures spatial continuity and inter-cluster metabolic heterogeneity and, where available, incorporated matched spatial transcriptomics-derived cell-type annotations for external validation. We also quantified the impact of preprocessing strategies on clustering performance. Through this comprehensive benchmark, we clarify the importance of preprocessing and method selection, identify clustering methods that perform robustly on spatial metabolomics data, and provide practical guidance for method selection. Finally, we developed an interactive online platform to help researchers choose appropriate clustering tools according to specific data characteristics and analytical objectives.

## 2. Materials and Methods

### 2.1. Data Collection and Preparation

To comprehensively assess the applicability of clustering methods to spatial metabolomics data, we assembled a benchmark comprising 12 MSI datasets from diverse sources (Table 1). These datasets span three major ion sources (AFADESI, MALDI, and DESI), four mass analyzers (Orbitrap, TOF, LTQ, and FT-ICR), and spatial resolutions ranging from 20 to 100 µm. The benchmark includes both publicly available datasets and internally generated datasets. Five datasets were collected from external sources, covering the major MSI ionization platforms commonly used in current research and representative tissue types including mouse brain, pig fetus, mouse fetus, and mouse kidney.

To further examine the effects of ionization mode and spatial resolution on clustering performance, we acquired MSI data from coronal serial sections of 7-week-old male mouse brains using the AFADESI-MSI platform in both positive and negative ion modes at spatial resolutions of 20 µm, 50 µm, and 100 µm. The spray solvent consisted of acetonitrile/water (80:20, *v*/*v*), and the AFADESI extraction gas flow rate was set to 45 L/min. Mass spectrometric detection was performed on a Q Exactive mass spectrometer (Thermo Fisher Scientific, Waltham, MA, USA) at a mass resolution of 70,000.

All raw data, whether experimentally generated or publicly obtained, were processed using a unified workflow in Cardinal (version 3.4.3, R package) [24] to minimize analytical bias. Peak picking was performed with the diff method in Cardinal, using a signal-to-noise ratio (SNR) threshold of 6 to identify local maxima as feature peaks. Peaks were then aligned across pixels with a tolerance of 10 ppm, and low-frequency features detected in fewer than 10% of pixels were removed. Peak areas were calculated after Gaussian smoothing of the raw spectra. Finally, SMAnalyst (available at: https://github.com/mzlab-research/SManalyst; accessed on 13/07/2025) [8] was used for quality control of the peak-picked spatial metabolomics data, including identification and removal of background regions.

To establish an objective biological validation framework and assess whether clustering results recapitulate anatomical structure, we used co-registered spatial metabolomics and spatial transcriptomics data from our previous SMIntegration study [25]. This paired dataset consists of spatial metabolomics data (mbrain2_pos50) and spatial transcriptomics (ST) data obtained from adjacent mouse brain sections. The ST data were generated using Stereo-seq (BGI Genomics, Shenzhen, China) at a native spatial resolution of 500 nm. To match the pixel resolution of the spatial metabolomics data, the transcriptomics data were aggregated to 50 µm by binning, and cell-type annotations were inferred by deconvolving the ST data with Cell2location (version 0.1.4) [26]. Precise co-registration between the spatial metabolomics and transcriptomics data was then performed using the SpatialData (version 0.2.5) framework [27]. This workflow enabled cell-type annotations to be mapped onto spatial metabolomics pixels, thereby providing an external biological reference for downstream evaluation of clustering performance.

**Table 1:** Summary of the 12 heterogeneous MSI datasets used in this study, covering multiple platforms and spatial resolutions.

Dataset Name	Ion Source	Mass Analyzer	Ionization Mode	Resolution (μm)	Sample Type	No. of Pixels	No. of *m*/*z*	Source
mbrain1_neg20	AFADESI	Orbitrap	Neg	20	Mouse brain	107,423	2005	In-house
mbrain1_neg50	AFADESI	Orbitrap	Neg	50	Mouse brain	23,531	2138	In-house
mbrain1_neg100	AFADESI	Orbitrap	Neg	100	Mouse brain	5875	2162	In-house
mbrain1_pos20	AFADESI	Orbitrap	Pos	20	Mouse brain	112,023	2578	In-house
mbrain1_pos50	AFADESI	Orbitrap	Pos	50	Mouse brain	22,792	2989	In-house
mbrain1_pos100	AFADESI	Orbitrap	Pos	100	Mouse brain	5373	3044	In-house
mbrain2_pos50	AFADESI	Orbitrap	Pos	50	Mouse brain	14,283	2654	In-house
PDX_mbrain_pos100	MALDI	FTICR	Pos	100	Mouse brain	3570	1131	[28]
pfetus_neg	DESI	LTQ	Neg	NA ^1^	Pig fetus	4959	687	[24]
mfetus_neg	MALDI	TOF	Neg	NA ^1^	Mouse fetus	16,197	2203	[29]
mbrain_neg40	MALDI	TOF	Neg	40	Mouse brain	32,368	2531	[30]
mkidney_neg40	MALDI	Orbitrap	Neg	40	Mouse kidney	45,623	258	[31]

^1^ Spatial resolution not explicitly reported in the original study.

### 2.2. Spatial Noise Score and Ion Filtering

MSI data contain complex matrix interferences and stochastic detection noise that manifest as random spatial distributions. Noisy ions interfere with the ability of clustering algorithms to identify tissue structures [22]. To systematically evaluate the impact of ion filtering on clustering results, we introduced the Spatial Noise Score (SNS) to characterize the spatial distribution properties of individual ions. The SNS quantifies the spatial noise level of a single ion image by computing the proportion of spatially dispersed pixels [32]. Specifically, the spatial intensity distribution of each ion is first binarized at the median value, and the proportion of “abnormal boundary pixels”—those whose labels are inconsistent with their neighboring pixels—is then calculated. A lower SNS value indicates greater spatial coherence of the ion’s distribution, suggesting it is more likely to represent a genuine anatomical region. To systematically evaluate the effect of noisy ion filtering on clustering algorithms, we employed a gradient filtering strategy, retaining the top 5%, 10%, 20%, 40%, 60%, 80%, and 100% of ions ranked by SNS from low to high (i.e., from low to high noise level) to construct test datasets. By comparing clustering performance across different filtering levels, this study aimed to identify the optimal preprocessing threshold that balances biological signal retention with noise suppression.

### 2.3. Clustering Methods

A total of 30 representative clustering methods were selected for systematic benchmarking. These methods are broadly categorized into spatially-aware methods and non-spatially-aware methods based on their algorithmic logic and their use of spatial information. Spatially-aware methods explicitly integrate pixel spatial coordinates with spectral similarity through architectures such as graph convolutional networks (GCN), graph attention mechanisms (GAT), or hidden Markov random fields (HMRF). Non-spatially-aware methods rely solely on metabolite expression profiles, and include commonly used community detection algorithms (e.g., Leiden and Louvain) as well as two-stage pipelines consisting of dimensionality reduction (PCA/t-SNE/UMAP) followed by conventional clustering (K-means/GMM/HC/Spectral).

The selected methods encompass algorithms specifically designed for MSI, advanced models transferred from spatial transcriptomics (ST), and general-purpose clustering methods used in bioinformatics analysis. Detailed descriptions of each algorithm are provided in Supplementary Note S1. To ensure a fair evaluation, all methods were run in a unified hardware environment. To improve the comparability of results across methods, the number of clusters was set to approximately the same value for all methods, with all remaining parameters set to their default values. The software versions and key parameter settings for all evaluated methods are provided in Supplementary Table S1. Table 2 summarizes the categorical attributes, application scenarios, technical architectures, and implementation languages of all evaluated methods.

### 2.4. Evaluation Metrics

#### 2.4.1. Spatial Continuity Assessment

Spatial continuity is an important metric for evaluating the performance of spatial clustering algorithms, with its core purpose being to assess whether clustering results exhibit coherence in their spatial distribution. This study employs the Percentage of Abnormal Spots (PAS) to quantify spatial continuity by calculating the degree of consistency between each pixel’s label and the labels of its neighboring pixels. PAS is particularly suitable for MSI clustering evaluation because it directly captures local label discontinuity and salt-and-pepper-like spatial fragmentation. We define PAS as the proportion of pixels whose cluster label is inconsistent with more than half of their 8 surrounding neighbors [22]. A lower PAS value indicates a lower degree of spatial fragmentation in the clustering results, resulting in smoother and more coherent tissue domain boundaries, which is consistent with the biological principle that metabolite distributions in the tissue microenvironment tend to exhibit regional continuity.

#### 2.4.2. Inter-Cluster Metabolic Heterogeneity Assessment

To quantify the correspondence between clustering regions and the spatial metabolic heterogeneity, we introduced the variance decomposition-based inter-cluster heterogeneity metric median-η^2^. This metric draws on the concept of between-group and within-group variance decomposition from analysis of variance, and is consistent with explanatory measures commonly used in spatial heterogeneity analysis [48], serving to quantify the extent to which cluster labels explain the variance in the spatial distribution of ions. For each ion feature in the dataset, we first compute the ratio of the between-cluster sum of squares to the total sum of squares (η^2^). The median of η^2^ values across all ions in the dataset is then taken as the composite evaluation score for that method. A median-η^2^ value closer to 1 indicates more pronounced differences in metabolic patterns across the clustering results.

#### 2.4.3. Computational Resource Efficiency Assessment

Computational efficiency and resource consumption are key metrics for evaluating the potential of algorithms for application to large-scale, high-resolution MSI data. This study primarily records the runtime and peak memory usage of each method during the clustering task. Runtime is measured as the total wall-clock time from algorithm initiation to final clustering output, recorded using Python’s system (Python 3.10.20 and R 4.5.0) time interface. Peak memory is measured by high-frequency sampling of the process’s resident set size (RSS), capturing the maximum memory footprint over the entire course of task execution. This dimension of evaluation is of considerable importance for identifying efficient algorithms capable of handling high-resolution spatial metabolomics data.

#### 2.4.4. Clustering Consistency and Biological Validation

To objectively evaluate the consistency of clustering results and their biological accuracy, this study uniformly employs Normalized Mutual Information (NMI) for quantitative assessment [49]. NMI is a commonly used statistical metric for measuring the similarity between two label partitions, with values ranging from 0 to 1, where higher values indicate greater concordance between the two partitions. In the experimental design, we first computed pairwise NMI between the clustering results of different methods on the same dataset to assess the clustering consistency of each algorithm on specific spatial metabolomics data. Subsequently, for datasets with adjacent Stereo-seq spatial transcriptomics sections, we used the cell-type annotations from the ST data as an external biological validation standard, computing the NMI between cluster labels and registered cell-type labels to evaluate the concordance of clustering results at the level of cellular composition.

### 2.5. Construction and Implementation of the Online Clustering Evaluation Platform

To lower the technical barrier for spatial metabolomics clustering analysis and improve the reproducibility of the evaluation workflow, we developed the online clustering evaluation platform SMcluster (available at: https://metax.genomics.cn/app/smcluster; accessed on 07/04/2026) based on the R Shiny framework. The platform is deployed on a high-performance cloud server equipped with 128 CPU cores and 1000 GB of memory, supporting concurrent multi-user access through a dynamic resource allocation mechanism. The platform is designed with ease of use as its core principle, implementing an end-to-end analysis pipeline that encompasses raw data import, ion quality filtering, batch execution of multiple algorithms, and automated metric evaluation. To facilitate rapid onboarding, the platform includes built-in standardized example datasets as well as detailed operational documentation. With respect to data security and privacy protection, the platform enforces strict controls: all data uploaded by users are processed exclusively in active memory during the session and are automatically destroyed upon session termination, ensuring that researchers retain full control and ownership of their raw data.

The platform requires input in the form of a standard CSV-format peak intensity matrix, in which the first two columns must explicitly define the spatial coordinates (X, Y) of each pixel, with subsequent columns corresponding to the detected intensities of individual ions. During the data import stage, the platform automatically identifies coordinate and feature columns and generates a data overview including pixel count, ion count, and coordinate range, while also providing a spatial distribution preview of target features to assist with preliminary quality inspection. In the preprocessing module, the platform integrates the SNS-based ion filtering strategy described in Section 2.2, allowing users to visually observe the effect of different filtering thresholds on ion image quality and to save filtering parameters to ensure the traceability of the analysis pipeline. In the core computation module, the platform fully supports all 30 clustering methods covered in Section 2.3. Users can configure multiple algorithms and parameter combinations within a unified session for queued batch execution, with the system automatically recording run logs and monitoring computational overhead. Upon task completion, the platform automatically computes key evaluation metrics including spatial continuity (PAS), inter-cluster metabolic heterogeneity (median-η^2^), clustering consistency (NMI), peak memory usage, and runtime (as detailed in Section 2.4), and presents the results in the form of interactive tables and figures.

## 3. Results

### 3.1. Effects of SNS-Based Ion Filtering on Spatial Clustering Performance

**Figure 1:**
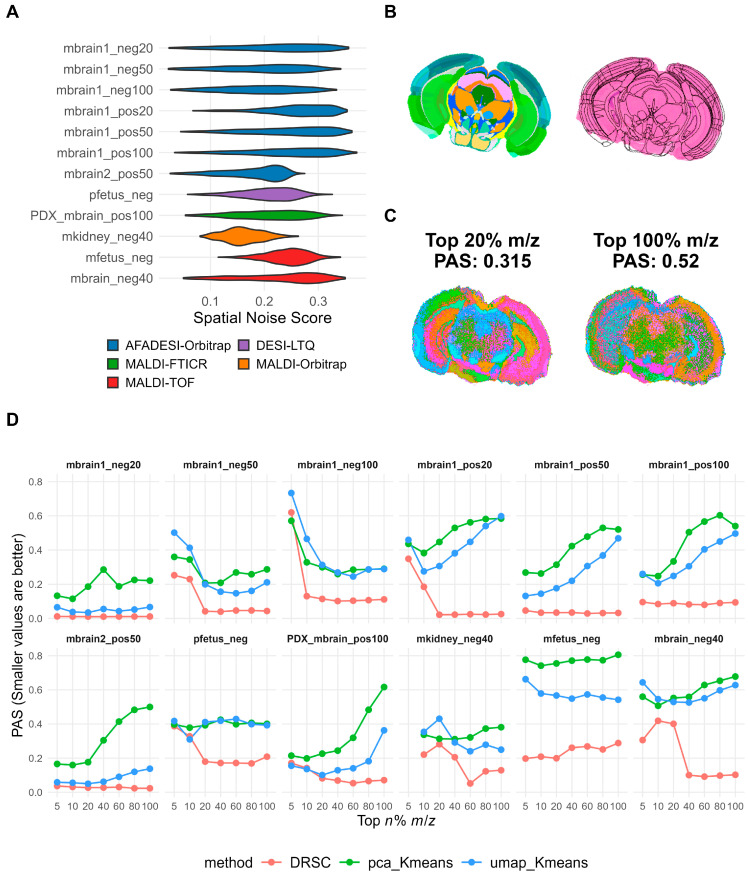
Effect of SNS-based ion filtering on clustering quality. (**A**) Distribution of SNS values for all ions across the 12 datasets. (**B**) H&E staining image of mouse brain tissue (mbrain1_pos50) and its corresponding structural annotations from the Allen Brain Atlas. (**C**) Comparison of clustering results produced by the pca_Kmeans algorithm with and without ion filtering. Different colors indicate distinct spatial clusters and are assigned independently. (**D**) PAS values of representative methods across all datasets at different ion filtering proportions (Top *n*% *m*/*z*).

Because MSI data are pervasively affected by technical noise and matrix interference, effective ion pre-filtering is essential for obtaining biologically meaningful clustering results. We therefore used the Spatial Noise Score (SNS) to quantify the spatial noise level of each ion and examined its distribution across the 12 heterogeneous datasets. Ions with high SNS values produced spatially fragmented ion images (Supplementary Figure S1). The overall SNS distributions varied substantially across platforms and tissue types: high-resolution AFADESI datasets contained a larger fraction of low-SNS, structurally informative ions, whereas several other datasets showed a higher proportion of noisy ions (Figure 1A).

To visually assess the effect of SNS-based ion filtering on clustering quality, we used the mouse brain dataset mbrain1_pos50 as an example and compared the results of the conventional non-spatially-aware method pca_Kmeans under different filtering thresholds. Without ion filtering, the clustering map displayed a pronounced salt-and-pepper pattern caused by large numbers of noisy ions, making tissue boundaries difficult to resolve (Figure 1C, right). In contrast, retaining only the top 20% highest-quality ions markedly improved spatial continuity, and the resulting substructures—including the cortex, hippocampus, and thalamus—showed better agreement with anatomical annotations from the Allen Brain Atlas (Figure 1B,C).

This trend was further supported by quantitative analysis. We examined how spatial continuity (PAS) changed with the ion-retention proportion for representative algorithms across all datasets (Figure 1D; Supplementary Figure S2). For the two non-spatially-aware methods pca_Kmeans and umap_Kmeans, PAS decreased substantially after moderate ion filtering (e.g., retaining the top 20%), indicating that removal of high-SNS ions improved their ability to recover spatially coherent structures. However, in some datasets, such as mbrain1_neg100 and mbrain1_pos20, overly stringent filtering (retaining only the top 5% or 10%) caused PAS to rise again. By contrast, spatially-aware methods such as DRSC maintained consistently low and stable PAS values across a wide range of ion-retention levels, indicating stronger robustness to noise. These results show that SNS-based ion filtering is a critical preprocessing step for non-spatially-aware methods. We therefore determined an appropriate filtering threshold for each dataset (Supplementary Table S2); at these thresholds, the PAS of non-spatially-aware methods decreased by an average of 27.67% relative to unfiltered data. All subsequent analyses were performed using the selected filtering thresholds.

### 3.2. Spatial Continuity Across Clustering Methods

**Figure 2:**
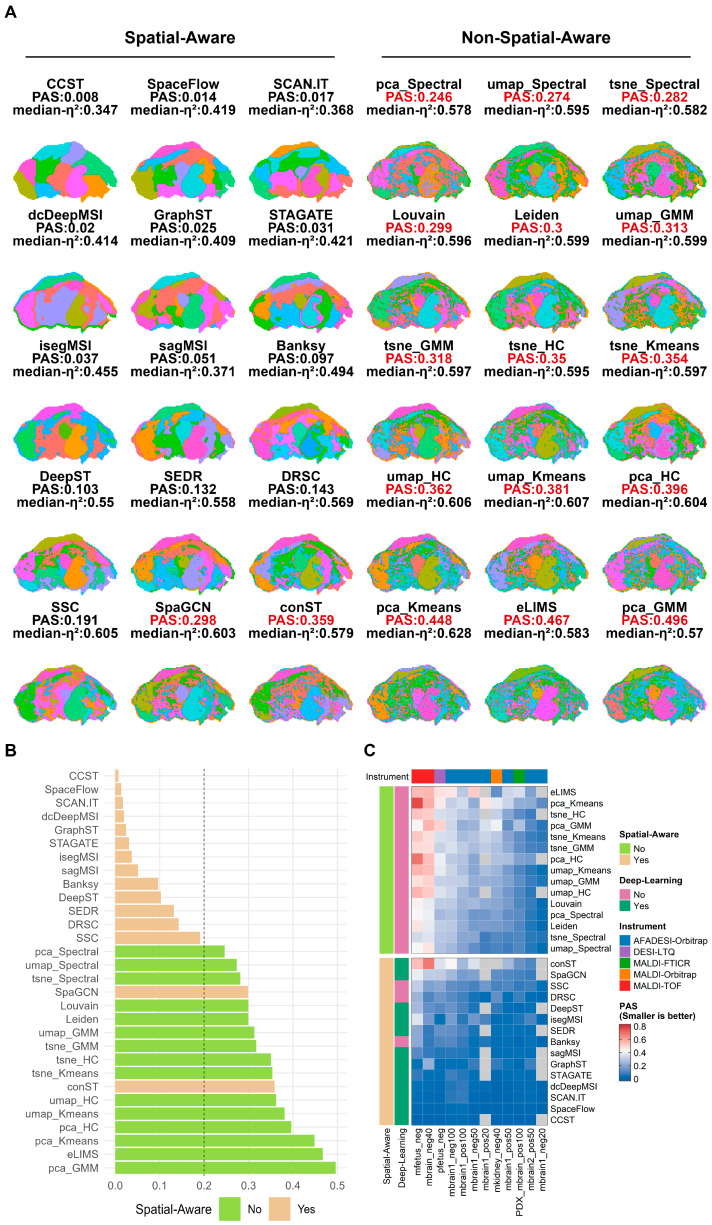
Spatial continuity (PAS) of clustering results from 30 clustering methods across different datasets. (**A**) Visualization of clustering results produced by 30 algorithms on the pfetus_neg dataset. Different colors indicate distinct spatial clusters and are assigned independently for each method. (**B**) PAS values of clustering results from each algorithm on the pfetus_neg dataset, ranked in descending order. (**C**) Heatmap of PAS scores for 30 methods across 12 datasets.

Identifying biologically coherent and meaningful spatial regions is the core task of spatial clustering; however, some clustering results exhibit fragmented clustering regions—the so-called salt-and-pepper effect—that fail to reveal true regional boundaries. To address this, we used the spatial continuity metric PAS to quantitatively evaluate the spatial clustering performance of all 30 algorithms on SNS-filtered data. In the pfetus_neg dataset, we observed that clustering regions with PAS > 0.2 exhibited pronounced fragmentation, whereas regions with PAS < 0.2 were relatively continuous with well-defined boundaries. Spatially-aware methods demonstrated a marked advantage: methods such as CCST, SCAN.IT, and SpaceFlow, by explicitly modeling spatial neighborhood dependencies, produced clustering maps with clear boundaries and low PAS values. In contrast, non-spatially-aware algorithms (e.g., K-means) were able to capture the approximate outlines of organs but were riddled with numerous isolated pixels (high PAS values), substantially reducing the anatomical interpretability of the spatial structures (Figure 2A,B).

Evaluation across all datasets further supported this observation: spatially-aware methods consistently exhibited low PAS values across data from different imaging platforms (MALDI, DESI, AFADESI), different resolutions (20–100 µm), and both positive and negative ion modes (Figure 2C; Supplementary Figure S3). Notably, in high-noise datasets such as mfetus_neg, the PAS values of non-spatially-aware methods increased sharply, while spatially-aware methods maintained good spatial continuity. These results indicate that the integration of spatial coordinates or neighborhood graph structures effectively suppresses random noise and produces continuous clustering regions.

To further evaluate whether PAS shows a consistent trend with an established alternative spatial metric, we compared PAS with Moran’s I [50] on the representative mbrain1_pos50 dataset. Moran’s I was calculated for each cluster using a one-vs-rest binary cluster-membership map, and the median value across clusters was used as the summary Moran’s I for each clustering result. PAS showed a clear negative correlation with the median Moran’s I, indicating that methods with lower spatial fragmentation according to PAS also tended to show higher Moran’s I values (Supplementary Figure S4A). This consistency supports the use of PAS as a simple and intuitive metric for evaluating spatial continuity in MSI clustering results.

### 3.3. Inter-Cluster Metabolic Heterogeneity Across Clustering Methods

**Figure 3:**
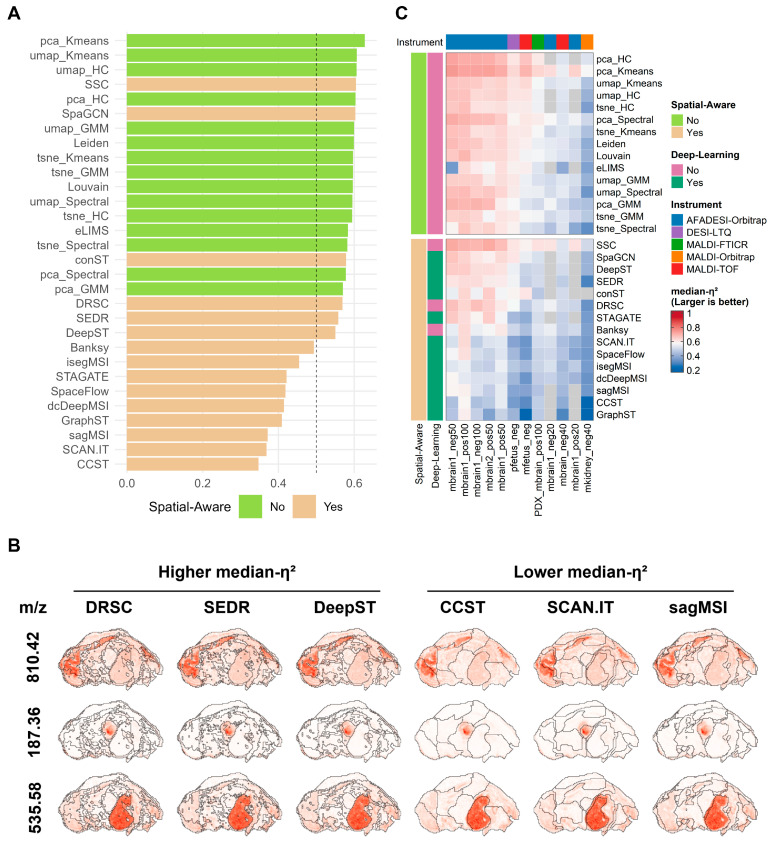
Comparison of the degree of ion heterogeneity between clustering regions. (**A**) Ranking of 30 algorithms by median-η^2^ on the pfetus_neg dataset. (**B**) Comparison of clustering regions from methods with high versus low median-η^2^ values against the spatial intensity distributions of organ marker ions. Black outlines denote cluster boundaries; red and white indicate high and low marker-ion intensities, respectively. (**C**) Heatmap of median-η^2^ values for clustering results of all methods across all datasets.

An ideal spatial clustering result should not only exhibit good spatial continuity, but should also minimize intra-cluster metabolic variation while maximizing inter-cluster metabolic differences. To this end, we employed median-η^2^ as an evaluation metric to quantify the degree of ion heterogeneity between clustering regions. Interestingly, the performance of clustering methods on this metric was not consistent with their performance on the PAS metric. Using the pfetus_neg dataset as an example, some methods that demonstrated strong spatial continuity in terms of PAS showed relatively low median-η^2^ values (Figure 3A), suggesting that an excessive emphasis on spatial continuity may obscure subtle metabolic differences. In contrast, non-spatially-aware methods such as pca_Kmeans, umap_Spectral, and Leiden achieved higher median-η^2^ scores, indicating greater ion heterogeneity between their clustering regions.

To further validate the biological relevance of median-η^2^, we compared the clustering regions of methods with high (>0.5) versus low (<0.5) median-η^2^ values against the spatial distributions of marker ions for specific organs (Figure 3B). The results show that for tissues including the midbrain (marker ion *m*/*z* 810.424), heart (marker ion *m*/*z* 187.36), and liver (marker ion *m*/*z* 537.11), the clustering regions identified by methods with higher median-η^2^ rankings showed greater concordance with the distribution of organ marker ions, whereas the clustering regions of methods with lower median-η^2^ values often failed to reflect the fine structural organization of tissue regions. This suggests that, in this dataset, the ability to accurately delineate tissue regions may be one of the factors contributing to greater inter-cluster heterogeneity and lower intra-cluster variation.

Comparing median-η^2^ values across all methods and datasets (Figure 3C), most algorithms achieved relatively high median-η^2^ values on AFADESI platform data, while values were generally lower on the mkidney_neg40 dataset, suggesting that median-η^2^ is to some extent influenced by the intrinsic ion heterogeneity structure of the data itself. Beyond dataset-level characteristics, there was also considerable variation among methods within the same dataset. For example, pca_Kmeans consistently achieved the highest median-η^2^ across all datasets. This stems from the properties of the method: PCA preferentially retains orthogonal directions of maximum variance, while K-means minimizes within-cluster sum of squares; together, these two components tend to produce compact within-cluster and well-separated between-cluster structures, which are precisely the type of clustering results that receive high median-η^2^ scores.

To further evaluate whether median-η^2^ is consistent with established alternative cluster-separation metrics, we compared median-η^2^ with the silhouette coefficient [51] and boundary silhouette coefficient [52] using clustering results generated by the 30 methods on the representative mbrain1_pos50 dataset. Median-η^2^ showed strong positive correlations with both alternative metrics (Supplementary Figure S4B,C), indicating that methods with higher variance-explained heterogeneity also tended to show better cluster separation. This consistency supports the use of median-η^2^ as an interpretable metric for evaluating inter-cluster metabolic heterogeneity in MSI clustering results.

### 3.4. Dual-Metric Evaluation Framework and Its Biological Validation

Both spatial continuity (PAS) and inter-cluster heterogeneity (median-η^2^) are important metrics for evaluating clustering results and must be considered jointly. However, the preceding results indicate that a trade-off exists between the two. For example, some spatially-aware methods (e.g., CCST) performed well on PAS (Figure 2B) but showed low median-η^2^ (Figure 3C), suggesting that an excessive reinforcement of spatial continuity may compromise the preservation of local metabolic differences. This study constructed a dual-metric filtering framework to identify methods that perform well on both spatial continuity and metabolic heterogeneity preservation.

The default thresholds were defined based on empirical observations and metric interpretation. Across the 12 datasets, clustering maps with PAS values below approximately 0.2 generally showed more continuous spatial regions and fewer salt-and-pepper-like fragmented pixels, whereas clustering results with PAS values above this level often exhibited obvious spatial discontinuity. Therefore, PAS < 0.2 was used as a default empirical threshold for spatial continuity. For median-η^2^, a value above 0.5 indicates that, for a typical ion, more than half of the total spatial variation can be explained by between-cluster differences. We therefore used median-η^2^ > 0.5 as a practical empirical threshold for meaningful inter-cluster metabolic heterogeneity. Based on these two default thresholds, the metric distributions and pass/fail outcomes for each dataset are shown in Supplementary Figures S5 and S6.

**Figure 4:**
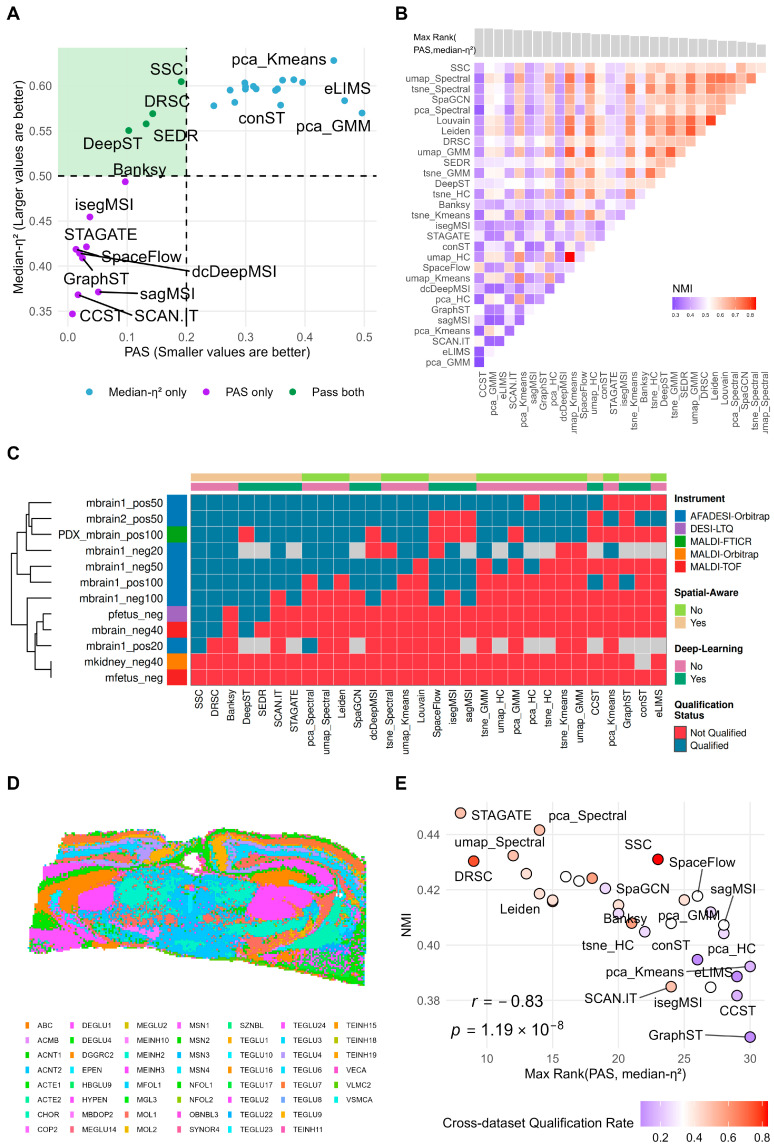
Dual-metric evaluation of spatial clustering results and cross-validation using spatial cell-type distribution data. (**A**) Distribution of clustering results from 30 methods on the two metrics (PAS and median-η^2^) for the pfetus_neg dataset. The green shaded area indicates the region passing both criteria: PAS < 0.2 and median-η^2^ > 0.5. (**B**) Heatmap of pairwise NMI between clustering results. (**C**) Summary of the number of algorithms passing PAS and median-η^2^ filtering across 12 datasets. (**D**) Spatial distribution of Stereo-seq cell-type annotation results from the adjacent section of the mbrain2_pos50 sample. (**E**) Correlation between the composite evaluation results and the biological validation evaluation (NMI between clustering results and cell-type annotations) for each method on the mbrain2_pos50 sample.

Using the pfetus_neg dataset as an example, 4 methods performed well on both spatial continuity and metabolic heterogeneity preservation, while 9 methods passed only on PAS and 17 passed only on median-η^2^ (Figure 4A). We further computed pairwise Normalized Mutual Information (NMI) between clustering results to assess the consistency among different clustering outcomes (Figure 4B). The results show that methods satisfying both metrics exhibited higher mutual consistency in their clustering results; this phenomenon was also observed in other datasets (Supplementary Figure S7), indicating that the clustering regions identified by these methods are more similar to one another compared to methods that fail to satisfy both criteria.

The pass rate statistics for all clustering methods across the 12 test datasets show that methods such as SSC and DRSC passed the dual-metric filtering on 9 datasets (pass rate 75%), demonstrating their ability to simultaneously achieve spatial continuity and inter-cluster heterogeneity across the majority of datasets (Figure 4C). In addition, some datasets—such as mfetus_neg—had very few or no methods that simultaneously passed both metrics. Examining the overall noise levels of each dataset revealed that datasets with lower pass rates tended to exhibit higher overall noise levels (Supplementary Figures S6 and S8).

We emphasize that these thresholds are empirical default cutoffs used only to convert the continuous PAS and median-η^2^ values into binary pass/fail outcomes for concise cross-dataset summarization. Within each dataset, changing the thresholds does not alter the raw PAS or median-η^2^ values, nor the relative ordering of methods based on these continuous metrics. However, the binary pass/fail status and cross-dataset pass counts may vary with threshold stringency. To assess robustness, we further compared strict, default, and relaxed dual-threshold settings. The results showed that although the absolute pass rates varied with threshold stringency, the overall trends of better- and worse-performing methods remained largely stable (Supplementary Figure S9).

To demonstrate the validity of the dual-metric approach for selecting clustering methods, we introduced cell-type annotations from adjacent Stereo-seq sections on the mbrain2_pos50 dataset (Figure 4D) as external biological validation, analyzing the concordance between MSI clustering results and ST cell-type annotations and comparing this concordance ranking with the dual-metric composite ranking (Figure 4E). The results show that methods ranked higher by the dual-metric composite evaluation exhibited greater concordance with cell-type annotations in the ST data, with a Spearman correlation coefficient of 0.83. The method with the best dual-metric composite ranking (STAGATE; NMI = 0.448) showed a 22% improvement in concordance with cell-type annotations (NMI) compared to the worst-ranked method (GraphST; NMI = 0.367). These results indicate that the dual-metric evaluation framework not only reflects the stable performance of algorithms on MSI data, but also captures, to a meaningful degree, their concordance with true tissue structures. In summary, this dual-metric evaluation framework can provide a more reliable basis for clustering method selection in subsequent spatial metabolomics research.

### 3.5. Computational Efficiency of Clustering Methods

To evaluate the computational efficiency of clustering methods in practical MSI data analysis, we systematically compared the runtime and peak memory usage of all methods across the 12 datasets (Supplementary Figure S10). The computational efficiency assessment was performed using the SNS-filtered input matrices used for the final clustering analysis. The 12 benchmark datasets contained 3570–112,023 pixels. All tests were conducted on the same workstation, mainly reflecting the practical performance of each method under its commonly recommended running environment. The workstation was equipped with 32 CPU cores, 64 GB of host memory, and one NVIDIA RTX 5070 Ti GPU. Non-deep-learning methods were run on the CPU, whereas deep-learning methods supporting GPU acceleration were run on the NVIDIA RTX 5070 Ti GPU.

The results showed substantial differences in computational cost among methods. Traditional methods based on PCA or graph-free clustering were generally the most efficient. For example, pca_Kmeans had the shortest average runtime, 1.1 s, and relatively low memory usage, 985.6 MB. pca_GMM, pca_HC, Leiden, and Louvain also showed high computational efficiency. UMAP-based pipelines provided a reasonable balance between speed and clustering flexibility, with an average runtime of approximately 73–74 s and average memory usage of approximately 1.68 GB.

In contrast, some deep-learning-based and graph-based methods required substantially greater computational resources. Under the same datasets and hardware conditions, dcDeepMSI had the longest average runtime, 2332.6 s. conST, DeepST, eLIMS, and t-SNE-based pipelines were also time-consuming on large-scale datasets. In terms of memory usage, GraphST showed the highest average peak memory consumption, 19.4 GB, followed by DeepST, SEDR, SpaGCN, and CCST. These results suggest that graph construction and message-passing architectures can become major computational bottlenecks for large-scale MSI datasets. Some methods relying on dense graph operations failed on high-resolution datasets because of out-of-memory errors.

Importantly, higher computational cost did not necessarily lead to better clustering performance. Methods such as SSC and DRSC showed strong performance under the dual-metric framework while maintaining moderate computational requirements, thereby achieving a favorable balance between clustering accuracy and computational efficiency. In contrast, some of the most computationally expensive methods did not consistently outperform lightweight methods. These results indicate that practical method selection for MSI studies should consider both clustering performance and computational cost.

### 3.6. Online Clustering Evaluation Platform

Given the substantial differences in ion noise distribution and biological heterogeneity across MSI datasets (Figure 1A), researchers in practice often need to optimize parameters and adjust strategies according to the specific characteristics of their data. To this end, we developed the interactive online evaluation platform SMcluster, designed to provide standardized tool support for clustering evaluation of MSI data. In SMcluster, PAS < 0.2 and median-η^2^ > 0.5 are provided as recommended default thresholds based on the benchmark datasets in this study, while users can flexibly customize these dual-metric thresholds according to the characteristics of their own datasets and analytical objectives.

To validate the reliability of the platform, we reproduced the evaluation workflow of this study on SMcluster, encompassing key steps including data upload, SNS-based ion filtering, and parallel multi-algorithm clustering (Supplementary Figure S11). In addition to the benchmark datasets described above, we further included a mouse uterine MSI dataset [53] as an independent non-brain demo dataset for platform testing. The methods that ranked among the top five in the original 12-dataset benchmark according to the dual-metric pass rate also showed favorable performance on this uterine dataset, achieving good spatial continuity and inter-cluster metabolic heterogeneity under the same evaluation framework (Supplementary Figure S12). This result is consistent with the main benchmark conclusions and further demonstrates that SMcluster can be applied to MSI datasets from tissue types beyond mouse brain. To further test the robustness of the evaluation conclusions, we compared on the platform the effect of different noise filtering intensities (retaining 80% versus 20% of ions) on clustering performance. The analysis results showed that as the number of retained ions increased, the spatial continuity (PAS) of non-spatially-aware methods deteriorated substantially (Supplementary Figure S13), while spatially-aware methods demonstrated strong noise robustness—a trend highly consistent with the conclusions of the benchmarking analysis in Section 3.1. In summary, the platform not only demonstrates the generalizability and reliability of the evaluation framework established in this study, but also provides researchers with a scientific basis for selecting optimal clustering strategies when confronted with complex and variable real-world data, through the provision of flexible personalized evaluation options such as freely adjustable noise filtering thresholds and dual-metric thresholds.

## 4. Discussion

In this study, we benchmarked 30 clustering algorithms across 12 heterogeneous mass spectrometry imaging (MSI) datasets. The main findings are as follows. Both preprocessing strategy and clustering method selection play critical roles in MSI-based spatial metabolomics analysis. Overall, spatially-aware methods generated more spatially continuous tissue regions, whereas SNS-based ion filtering markedly improved the performance of non-spatially-aware clustering methods. Among the evaluated algorithms, SSC and DRSC achieved a favorable balance between low spatial fragmentation and high inter-cluster metabolic heterogeneity. In addition, the SMcluster platform developed in this study provides practical support for selecting appropriate clustering methods for specific MSI datasets.

SNS-based ion filtering had a pronounced effect on non-spatially-aware methods, which is consistent with the noise characteristics of MSI data. Noisy ions with spatially fragmented distributions can dominate distance calculations in feature space, thereby leading to salt-and-pepper-like clustering results. Removing high-SNS ions therefore improves the ability of conventional clustering methods to recover coherent tissue structures. However, overly stringent filtering may also remove informative ions, indicating that filtering thresholds should be optimized according to dataset-specific characteristics. Spatially-aware methods were less sensitive to ion filtering, probably because they incorporate spatial coordinates or neighborhood relationships, which can partially suppress random pixel-level fluctuations.

A central finding of this benchmark is the trade-off between spatial continuity and metabolic heterogeneity, which motivated the construction of the dual-metric evaluation framework. Clustering results with high spatial continuity did not necessarily show large metabolic differences between cluster labels, suggesting that excessive spatial smoothing may obscure local metabolic differences. Conversely, methods that produced clusters with strong metabolic separation were more likely to generate spatially fragmented clustering maps. Therefore, a dual-metric framework is needed to evaluate whether clustering results simultaneously preserve spatial coherence and metabolic distinction. Across multiple datasets, SSC and DRSC showed high pass rates under the dual-metric criteria. Moreover, methods ranked highly by the dual-metric evaluation tended to produce more consistent clustering results. In the dataset with matched cell-type annotations, the dual-metric ranking was also strongly correlated with the validation results based on spatial transcriptomics (ST)-derived cell-type labels, further supporting the biological relevance of this framework.

From a practical perspective, the stability of clustering algorithms should be considered together with computational cost. Some deep-learning-based and graph-based clustering methods required substantially greater runtime and memory, but did not consistently outperform lightweight methods. In contrast, methods such as SSC and DRSC showed strong dual-metric performance across multiple datasets while maintaining moderate computational requirements. This indicates that practical method selection should take into account data size, noise level, hardware availability, and analytical objectives. The SMcluster platform developed in this study integrates ion filtering, multi-algorithm clustering, metric evaluation, and customizable thresholds into a standardized analysis workflow, thereby providing convenient support for clustering method screening and evaluation.

This study has several limitations. First, although the benchmark included datasets from multiple detection platforms and tissue types, additional disease models, organ samples, and acquisition conditions should be incorporated in future validation studies. Second, to ensure comparability, most algorithms were run using default or recommended parameters; dataset-specific parameter tuning may further improve the performance of some methods. Third, external biological validation was performed using only one paired MSI-ST dataset. The inclusion of more paired multi-omics datasets in future studies will help further evaluate the biological accuracy of MSI clustering algorithms.

## 5. Conclusions

This study systematically benchmarked 30 clustering methods across 12 heterogeneous MSI datasets and proposed a dual-metric framework for evaluating spatial continuity and inter-cluster metabolic heterogeneity. The results demonstrate that SNS-based ion filtering improves the performance of non-spatially-aware methods, while spatially-aware methods are generally more robust to noisy ions. Among the evaluated algorithms, SSC and DRSC showed robust performance across datasets by achieving a favorable balance between low spatial fragmentation and high metabolic heterogeneity. Biological validation using spatial transcriptomics-derived cell-type annotations further supported the relevance of the dual-metric evaluation framework. Finally, the SMcluster platform implements the complete workflow for ion filtering, clustering, and evaluation, providing a practical tool for method selection in MSI-based spatial metabolomics studies. Together, this benchmark provides standardized guidance for spatial clustering analysis and highlights the need to jointly consider preprocessing, clustering performance, and computational feasibility.

## Figures and Tables

**Table 2:** Summary of the evaluated clustering algorithms.

Algorithm	Primary Application	Spatially-Aware	Deep Learning	Language	Reference
Banksy	Spatial transcriptomics	✓	✕	Python	[33]
CCST	Spatial transcriptomics	✓	✓	Python	[34]
conST	Spatial transcriptomics	✓	✓	Python	[35]
dcDeepMSI	Spatial metabolomics	✓	✓	Python	[36]
DeepST	Spatial transcriptomics	✓	✓	Python	[37]
DRSC	Spatial transcriptomics	✓	✕	R	[38]
eLIMS	Spatial metabolomics	✕	✕	Python	[20]
GraphST	Spatial transcriptomics	✓	✓	Python	[39]
isegMSI	Spatial metabolomics	✓	✓	Python	[19]
Leiden	Single-cell omics	✕	✕	R/Python	[40]
Louvain	Single-cell omics	✕	✕	R/Python	[41]
pca_GMM	General	✕	✕	R/Python	--
pca_HC	General	✕	✕	R/Python	--
pca_Kmeans	General	✕	✕	R/Python	--
pca_Spectral	General	✕	✕	R/Python	--
sagMSI	Spatial metabolomics	✓	✓	Python	[12]
SCAN.IT	Spatial transcriptomics	✓	✓	Python	[42]
SEDR	Spatial transcriptomics	✓	✓	Python	[43]
SpaceFlow	Spatial transcriptomics	✓	✓	Python	[44]
SpaGCN	Spatial transcriptomics	✓	✓	Python	[45]
SSC	Spatial metabolomics	✓	✕	R	[11]
STAGATE	Spatial transcriptomics	✓	✓	Python	[13]
tsne_GMM	General	✕	✕	R/Python	[46]
tsne_HC	General	✕	✕	R/Python	--
tsne_Kmeans	General	✕	✕	R/Python	[46]
tsne_Spectral	General	✕	✕	R/Python	--
umap_GMM	General	✕	✕	R/Python	[47]
umap_HC	General	✕	✕	R/Python	--
umap_Kmeans	General	✕	✕	R/Python	[47]
umap_Spectral	General	✕	✕	R/Python	--

*Note:* “--” indicates that the method is a conventional baseline combination of standard dimensionality-reduction and clustering algorithms rather than a standalone published method. Analyses were run using Python 3.10.20 and R 4.5.0.

## Data Availability

The data presented in this study are openly available in [OMIX database of the National Genomics Data Center] at [https://ngdc.cncb.ac.cn/omix/release/OMIX016317], reference number [OMIX016317]. The SMcluster online platform is available at https://metax.genomics.cn/app/smcluster. The source code and Docker image for local deployment are available at https://github.com/luyunning/SMcluster.

## Supplementary Materials

The following supporting information can be downloaded at: https://www.mdpi.com/article/10.3390/metabo16050348/s1, Supplementary Note S1: Detailed descriptions of clustering algorithms [54,55]; Supplementary Table S1, key parameter settings for clustering methods; Supplementary Table S2, dataset-specific Spatial Noise Score (SNS) filtering thresholds; Supplementary Table S3, η^2^ values of organ-specific marker ions in the pfetus_neg dataset; Supplementary Figure S1, Spatial Distributions of Ions Near SNS Cutoff Thresholds; Supplementary Figure S2, Spatial Clustering Maps under Different SNS Filtering Proportions; Supplementary Figure S3, Spatial Clustering Maps Generated by All Evaluated Methods; Supplementary Figure S4, Validation of PAS and median-η^2^ Using Alternative Spatial Continuity and Cluster-Separation Metrics; Supplementary Figure S5, Joint Distributions of PAS and median-η^2^ for All Methods Across Datasets; Supplementary Figure S6, Pass Rates of Each Method under Dual Criteria of PAS and median-η^2^ Across Datasets; Supplementary Figure S7, Pairwise Normalized Mutual Information (NMI) Between Clustering Results of Different Methods; Supplementary Figure S8, Distributions of Spatial Noise Score (SNS) in Filtered Datasets Used for Clustering; Supplementary Figure S9, Robustness of Dual-Metric Evaluation under Different Threshold Stringencies; Supplementary Figure S10, Computational Efficiency of Clustering Methods Across Datasets; Supplementary Figure S11, Workflow Reproduction and Benchmarking Results on the SMcluster Online Platform; Supplementary Figure S12, Evaluation of an Independent Mouse Uterine MSI Dataset; Supplementary Figure S13, Comparison of Clustering Results under Different SNS Filtering Stringencies: Retaining the Top 20% versus 80% of Ions.

## Abbreviations

The following abbreviations are used in this manuscript:

SNS	Spatial Noise Score
PAS	Percentage of Abnormal Spots
AFADESI	Air flow-assisted desorption electrospray ionization
MSI	Mass spectrometry imaging
