# Supplementary material for: Benchmarking Spatial Clustering Methods for Mass Spectrometry-Based Spatial Metabolomics
Source: Metabolites. 2026 May 21;16(5):348. doi: 10.3390/metabo16050348 (PMC13208584; doi:10.3390/metabo16050348)

Supplementary Materials

Benchmarking Spatial Clustering Methods for Mass Spectrometry-Based Spatial Metabolomics

Yunning Lu [1,†], Zhanlong Mei [2,†], Haoke Deng [2], Yun Zhao [2], Chunlu Feng [2] and Siqi Liu [2,*]

[1] School of Biology and Biological Engineering, South China University of Technology, Guangzhou 510006, China; 202320148479@mail.scut.edu.cn
[2] BGI Genomics, Shenzhen 518083, China; meizhanlong@genomics.cn (Z.M.); denghaoke@genomics.cn (H.D.); zhaoyun@genomics.cn (Y.Z.); fengchunlu@genomics.cn (C.F.)
[*] Correspondence: siqiliu@genomics.cn; Tel.: +86-139-1002-1096
[†] These authors contributed equally to this work.

-Supplementary Note

Note S1: Detailed Descriptions of Clustering Algorithms

-Supplementary Tables

Table S1: Key Parameter Settings for Clustering Methods

Table S2: Dataset-Specific Spatial Noise Score (SNS) Filtering Thresholds

Table S3: η^2 of Organ-Specific Marker Ions in the pfetus_neg Dataset

-Supplementary Figures

Figure S1: Spatial Distributions of Ions Near SNS Cutoff Thresholds

Figure S2: Spatial Clustering Maps under Different SNS Filtering Proportions

Figure S3: Spatial Clustering Maps Generated by All Evaluated Methods

Figure S4: Validation of PAS and median-η^2 Using Alternative Spatial Continuity and Cluster-Separation Metrics

Figure S5: Joint Distributions of PAS and median-η^2 for All Methods Across Datasets

Figure S6: Pass Rates of Each Method under Dual Criteria of PAS and median-η^2 Across Datasets

Figure S7: Pairwise Normalized Mutual Information (NMI) Between Clustering Results of Different Methods

Figure S8: Distributions of Spatial Noise Score (SNS) in Filtered Datasets Used for Clustering

Figure S9: Robustness of Dual-Metric Evaluation under Different Threshold Stringencies

Figure S10: Computational Efficiency of Clustering Methods Across Datasets

Figure S11: Workflow Reproduction and Benchmarking Results on the SMcluster Online Platform

Figure S12: Evaluation of an Independent Mouse Uterine MSI Dataset

Figure S13: Comparison of Clustering Results under Different SNS Filtering Stringencies: Retaining the Top 20% versus 80% of Ions

Note S1: Detailed Descriptions of Clustering Algorithms

1. Spatially Aware Clustering Methods

1.1 Algorithms Specifically Developed for Mass Spectrometry Imaging (MSI)

dc-DeepMSI adopts a divide-and-conquer deep learning strategy that decouples MSI image segmentation into two independent subtasks, namely dimensionality reduction and feature clustering. Specifically, the method first employs an autoencoder to extract spectral features and subsequently uses a temporally ensembled convolutional neural network (CNN) to capture spatial contextual information [36]. In this study, we evaluated its spat-contig mode, which is optimized for spatially contiguous regions.

SagMSI constructs a spatially aware graph that integrates both spatial proximity and spectral similarity, and then aggregates neighborhood features through a graph convolutional network (GCN). Its major advantage lies in enabling noise-robust, boundary-preserving pixel-level segmentation [12].

SSC, implemented in the Cardinal R package, is a probabilistic modeling framework that extends the nearest shrunken centroids algorithm to the spatial domain. It enhances clustering coherence by incorporating either Gaussian-weighted spatially aware distance (SA) or structurally adaptive spatially aware distance (SASA) [11].

iSegMSI adopts a regularization-based segmentation strategy. In this study, we used its fully unsupervised mode, which optimizes an objective function to preserve spatial structural integrity while performing feature dimensionality reduction [19].

1.2 Algorithms Transferred from Spatial Transcriptomics (ST)

STAGATE employs a graph attention autoencoder (GAT) to adaptively learn similarity weights among neighboring pixels, thereby enabling accurate delineation of tissue boundaries [13].

GraphST enhances local structural discrimination through graph self-supervised contrastive learning by minimizing the embedding distance between adjacent spots on a spatial k-nearest-neighbor graph [39].

SpaGCN integrates spatial coordinates, spectral features, and optionally histological similarity into a weighted undirected graph, and then aggregates information through graph convolutional layers [45].

SEDR combines a masked autoencoder with a variational graph autoencoder (VGAE) to learn latent representations that incorporate spatial information by reconstructing randomly masked expression vectors [43].

DeepST [37] and conST [35] employ a variational graph autoencoder and a contrastive learning framework, respectively, with the aim of capturing multimodal features through advanced graph representation learning.

SpaceFlow introduces a spatial regularization loss to generate spatially coherent embeddings and model pseudo-spatiotemporal trajectories [44].

SCAN-IT uses a geometry-aware α-complex graph to capture local microenvironments [42].

CCST learns node embeddings that encode spatial location by training a Deep Graph Infomax (DGI) network [34].

Among non-deep-learning methods, DR-SC performs dimensionality reduction and clustering within a unified framework, using a Potts model prior to ensure boundary smoothness [38]. BANKSY, by contrast, enhances intrinsic cellular transcriptomic features using azimuthal Gabor filters (AGF) and enables a transition from cell-type-level clustering to tissue-domain-level clustering by adjusting the parameter λ [33].

2. Non-spatial Baseline Methods

2.1 Graph-based Community Detection Algorithms

The Leiden [40] and Louvain [41] algorithms are widely used clustering strategies in single-cell omics. These methods first construct a shared nearest-neighbor (SNN) graph based on the spectral expression matrix and subsequently identify tightly connected groups of pixels by maximizing modularity. Because their algorithmic framework relies entirely on spectral similarity without incorporating spatial coordinate information, they are commonly used as baseline references for assessing the contribution of spatial information to clustering performance.

2.2 Two-stage Dimensionality Reduction–Clustering Pipelines

In this study, we systematically evaluated 12 commonly used "dimensionality reduction + clustering" combinations in MSI analysis to cover the benchmark performance of conventional analytical workflows. In the first stage, the high-dimensional metabolite

matrix was projected into a low-dimensional latent space using principal component analysis (PCA), t-distributed stochastic neighbor embedding (t-SNE) [54], or uniform manifold approximation and projection (UMAP) [47], with the aim of reducing feature redundancy.

In the second stage, clustering was performed in the resulting low-dimensional space using four classical algorithms: k-means clustering (K-means), Gaussian mixture model (GMM), hierarchical clustering (HC), and spectral clustering [55]. These combinations, such as PCA-Kmeans, tSNE-GMM, and UMAP-Spectral, represent the optimal classification capacity of metabolic features in the absence of spatial constraints and therefore constitute the core baselines in this benchmark study.

2.3 Non-spatial Ensemble Learning Framework

eLIMS repeatedly applies UMAP to randomly sampled feature subsets to generate multiple low-dimensional embeddings. For each embedding, a Gaussian mixture model is used to determine cluster centers, which are then used to initialize k-means clustering. Finally, the clustering results from all runs are integrated through majority voting to produce the final segmentation [20]. Although this method was specifically designed for MSI, it is classified here as a non-spatially aware ensemble baseline because no explicit spatial neighborhood constraints among pixels are incorporated during the integration process.

Table S1. Key Parameter Settings for Clustering Methods.

Methods	Versions	Key parameters
Banksy	0.1.6	--lambda 0.8 --k_geom 15
CCST	†	--Dim_PCA 200 --lambda_I 0.3 --k_neighbors 20
conST	1.4	--cell_feat_dim 300 --k 10
dcDeepMSI	†	--stepsize_tv 1 --mode spat-contig
DeepST	2.0.2	--k_neighbors 15 --pca_n_comps 200
DRSC	3.7	--q 50
eLIMS	†	--n_chunks 5
pca_GMM	§	--n_components 30
pca_HC	§	--n_components 30 --linkage "ward" --metric "Euclidean"
pca_Kmeans	§	--n_components 30
pca_Spectral	§	--n_components 30 --n_neighbors 10
tsne_GMM	§	--n_components 3
tsne_HC	§	--n_components 3 --linkage "ward" --metric "Euclidean"
tsne_Kmeans	§	--n_components 3
tsne_Spectral	§	--n_components 3 --n_neighbors 10
umap_GMM	§	--n_components 3
umap_HC	§	--n_components 3 --linkage "ward" --metric "Euclidean"
umap_Kmeans	§	--n_components 3
umap_Spectral	§	--n_components 3 --n_neighbors 10
GraphST	1.1.1	--n_neighbors 5
isegMSI	†	--stepsize_sim 1 --stepsize_con 1
Leiden	‡	--n_pcs 50 --n_neighbors 15
Louvain	‡	--n_pcs 50 --n_neighbors 15
sagMSI	†	--sim_threshold 0.84
SCAN-IT	0.1	--knn_n_neighbors 15 --Dim_PCA 300
SEDR	1.0.0	--k 20 --cell_feat_dim 200
SpaceFlow	1.0.3	--spatial_regularization_strength 1 --n_neighbors 10
SpaGCN	1.2.7	--p 0.5
SSC	3.4.3	--r 2 --s 2
STAGATE	1.0.0	--k_cutoff 20

§ Implemented using scikit-learn v1.5.2 (PCA, t-SNE, KMeans, AgglomerativeClustering, SpectralClustering, GaussianMixture) and umap-learn v0.5.7. ‡ Performed via Scanpy v1.11.1 (Leiden: leidenalg v0.10.2; Louvain: louvain v0.8.2). † No version tag available; source code obtained from the original GitHub repository.

Table S2. Dataset-Specific Spatial Noise Score (SNS) Filtering Thresholds.

Data	Target clusters number	SNS filter cutoff (Top N%)	PAS decreased by (%; pca_Kmeans)	PAS decreased by (%; umap_Kmeans)
mbrain1_neg20	30	20	15.88	48.26
mbrain1_neg50	30	20	27.69	5.61
mbrain1_neg100	30	20	-3.16	-8.42
mbrain1_pos20	30	20	23.56	48.67
mbrain1_pos50	30	20	39.49	62.18
mbrain1_pos100	30	20	38.1	49.76
mbrain2_pos50	30	20	64.76	63.87
pfetus_neg	10	20	2.11	-4.78
PDX_mbrain_pos100	10	20	63.32	72.2
mkidney_neg40	7	60	15.69	3.7
mfetus_neg	20	20	6.31	-4.58
mbrain_neg40	30	40	17.61	16.23

Table S3. η^2 of Organ-Specific Marker Ions in the pfetus_neg Dataset.

m/z	DRSC	SEDR	DeepST	CCST	SCAN. IT	sagMSI
810.424	0.627	0.62	0.632	0.335	0.327	0.379
187.36	0.574	0.497	0.511	0.325	0.372	0.415
537.11	0.731	0.714	0.747	0.58	0.637	0.584

Figure S1. Spatial Distributions of Ions Near SNS Cutoff Thresholds.

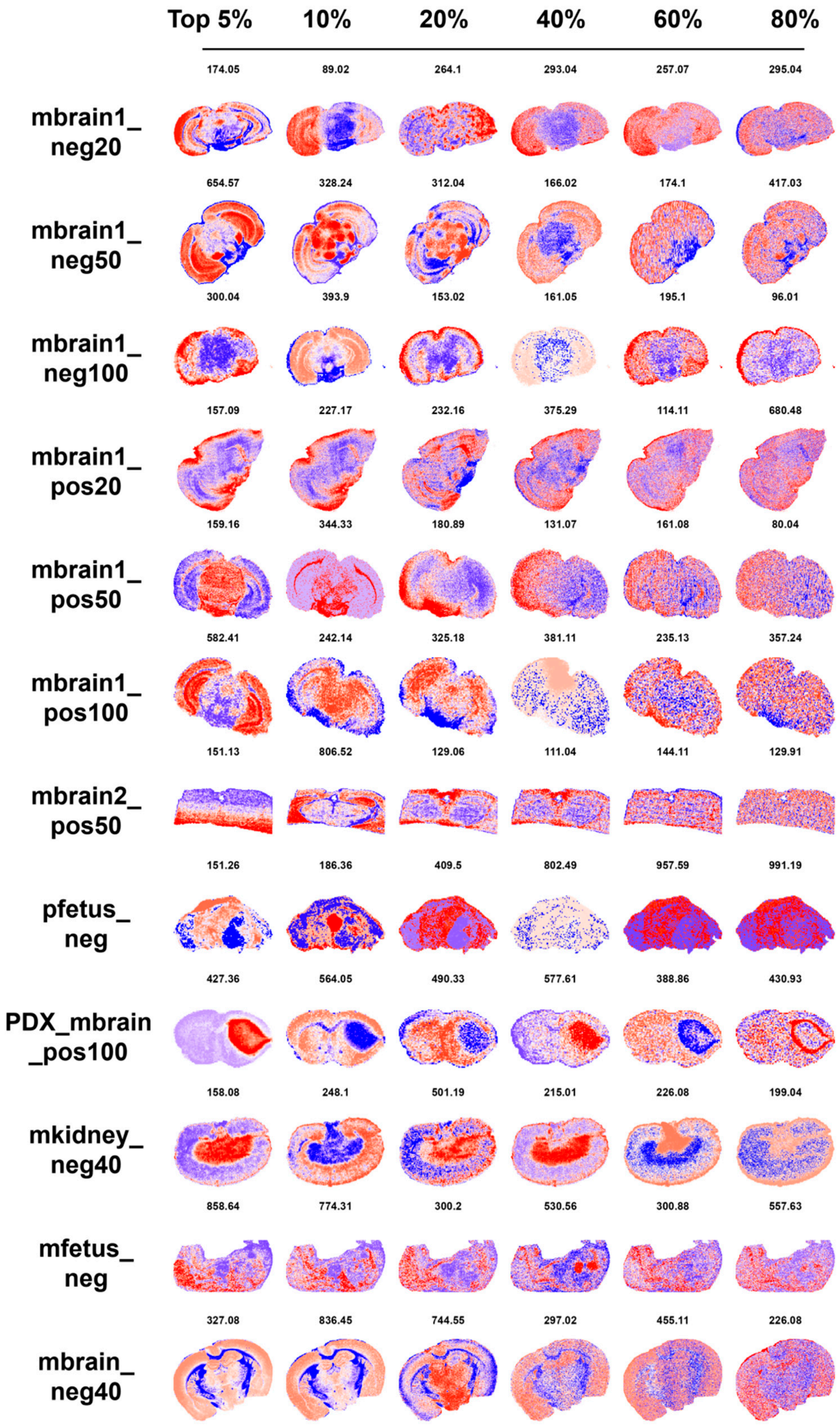

Figure S2. Spatial Clustering Maps under Different SNS Filtering Proportions.

A. mbrain1_neg20

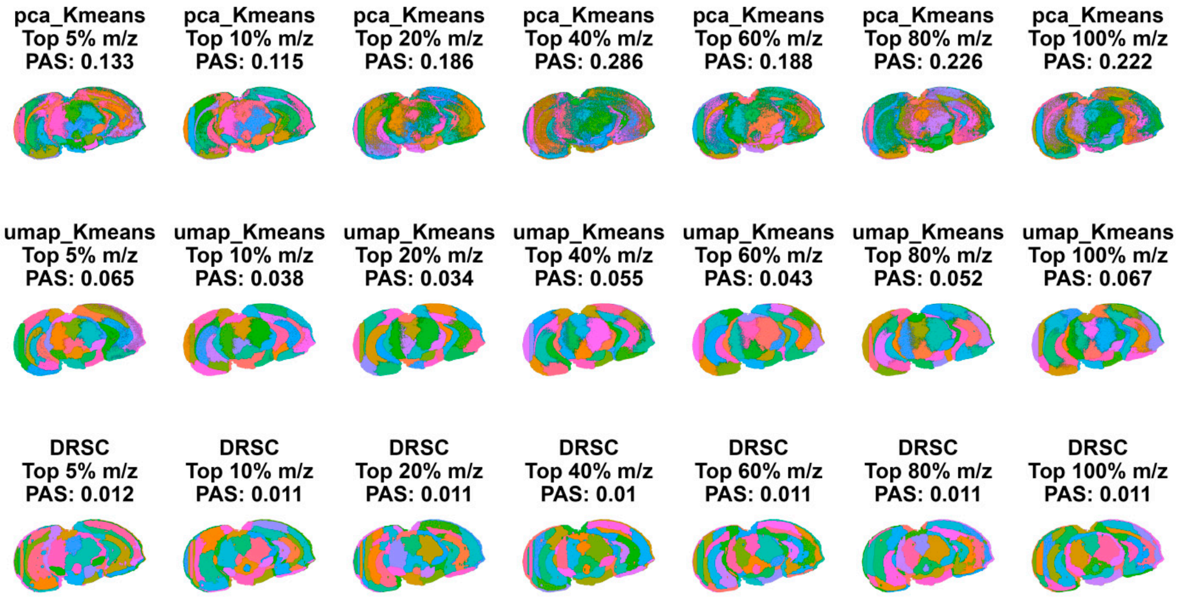

B. mbrain1_neg50

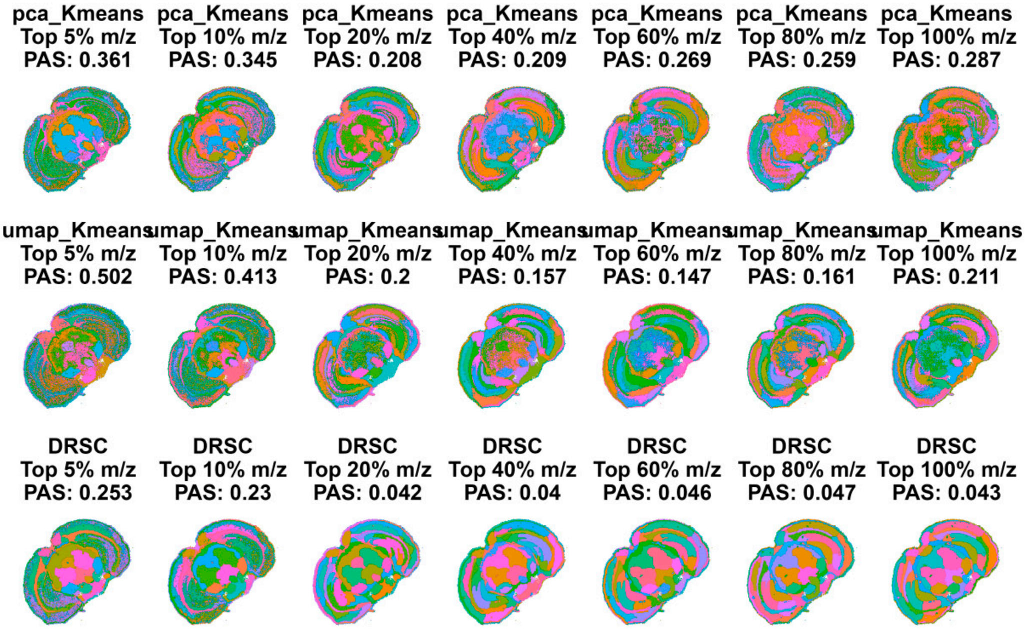

C. mbrain1_neg100

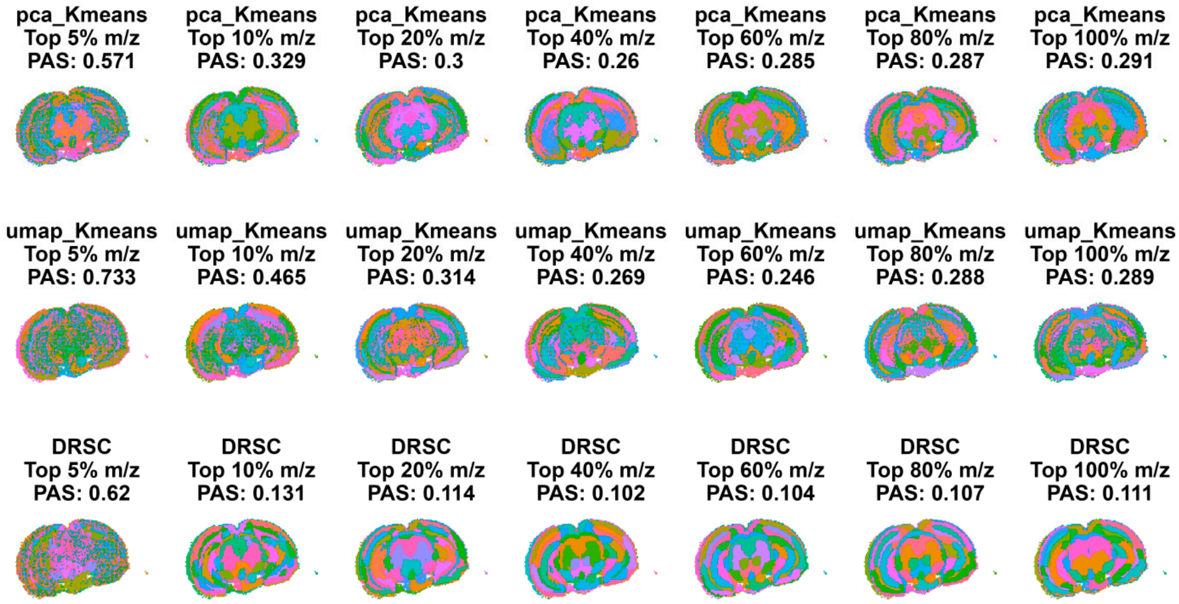

D. mbrain1_pos20

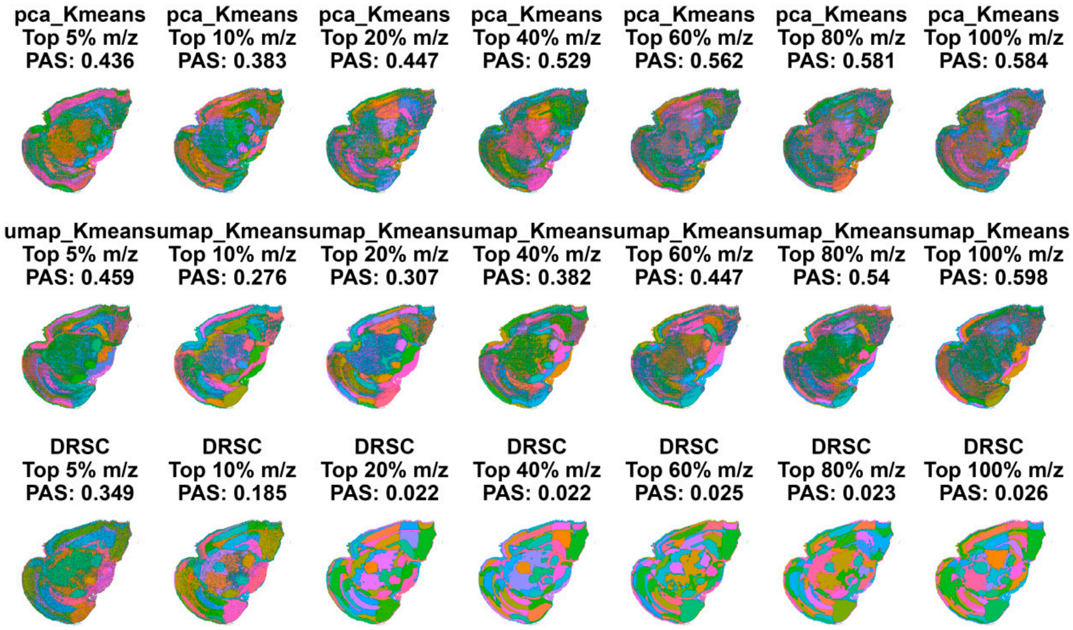

E. mbrain1_pos50

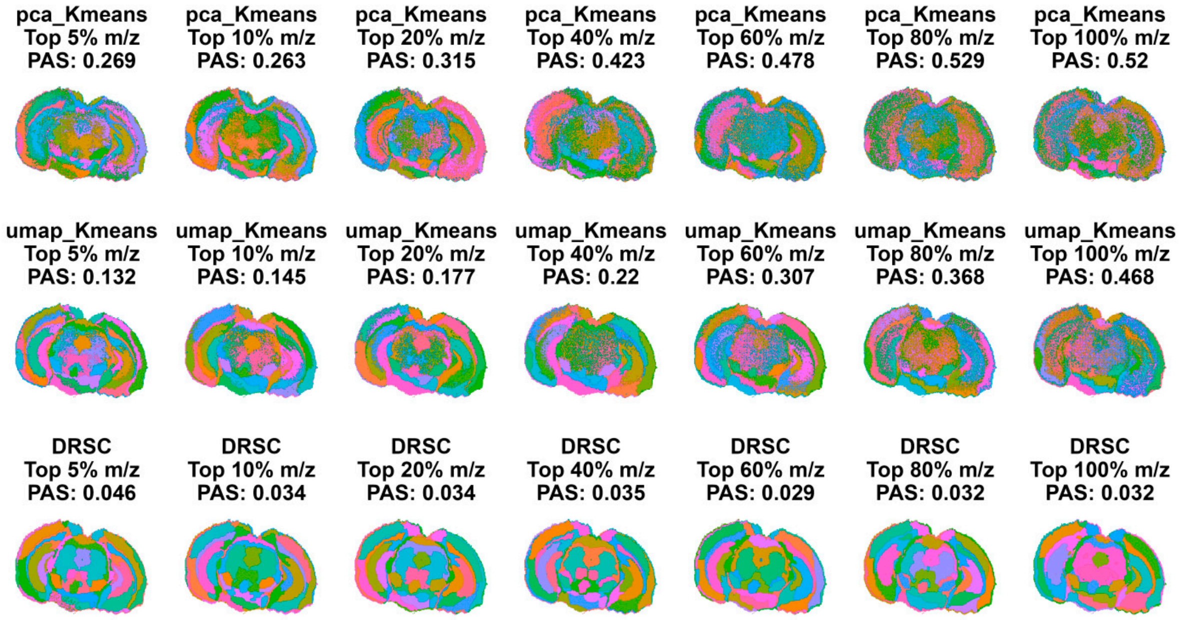

F. mbrain1_pos100

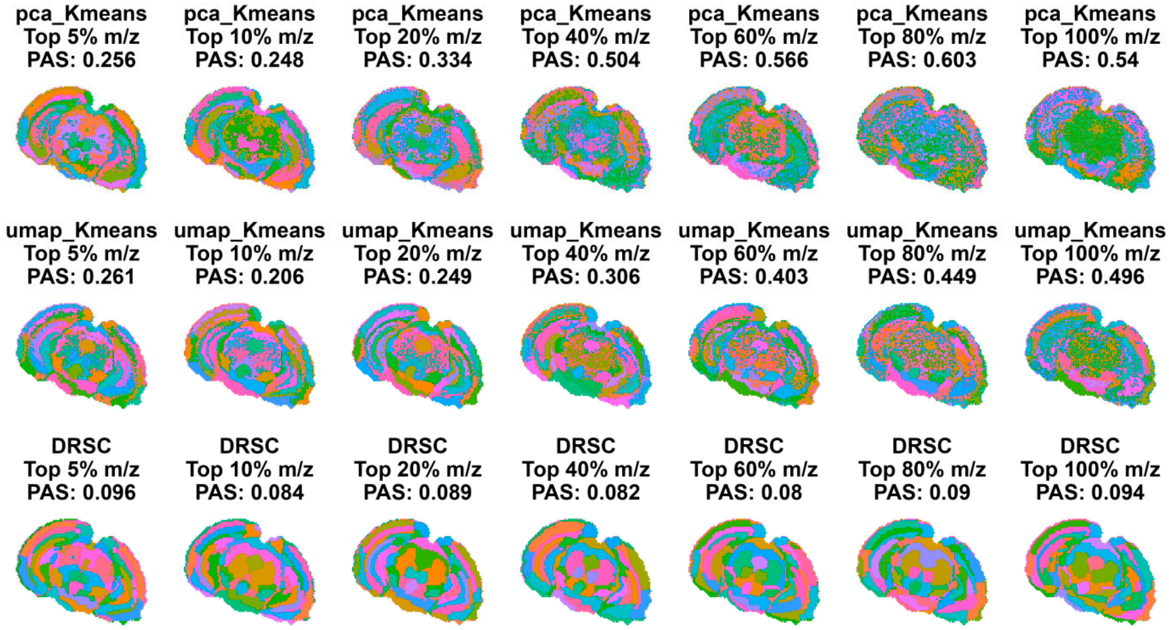

G. mbrain2_pos50

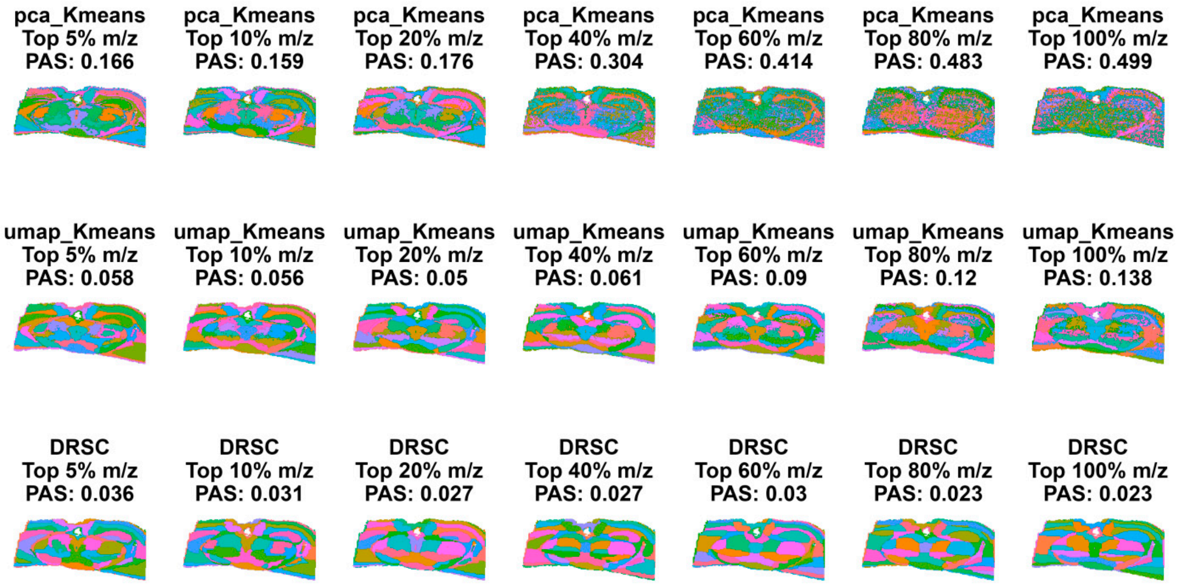

H. pfetus_neg

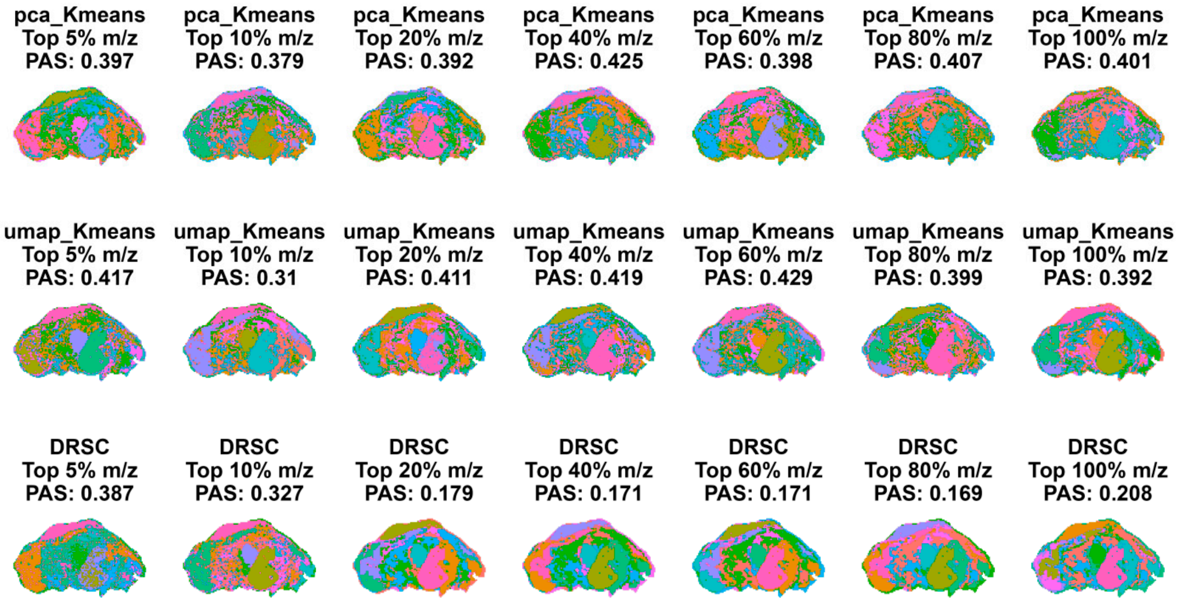

I. PDX_mbrain_pos100

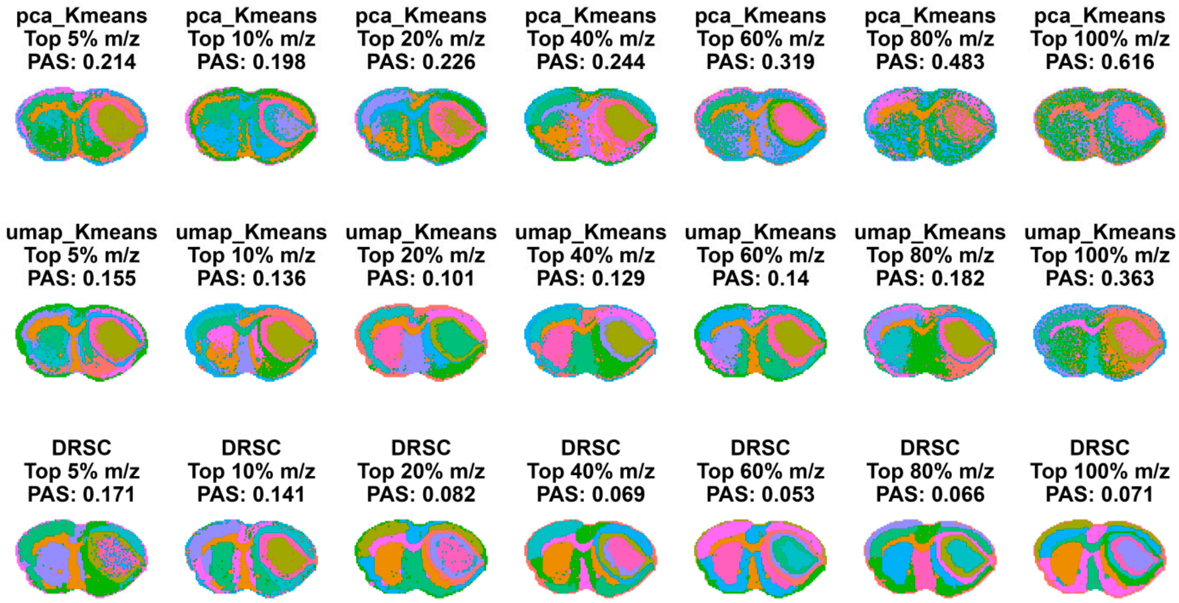

J. mkidney_neg40

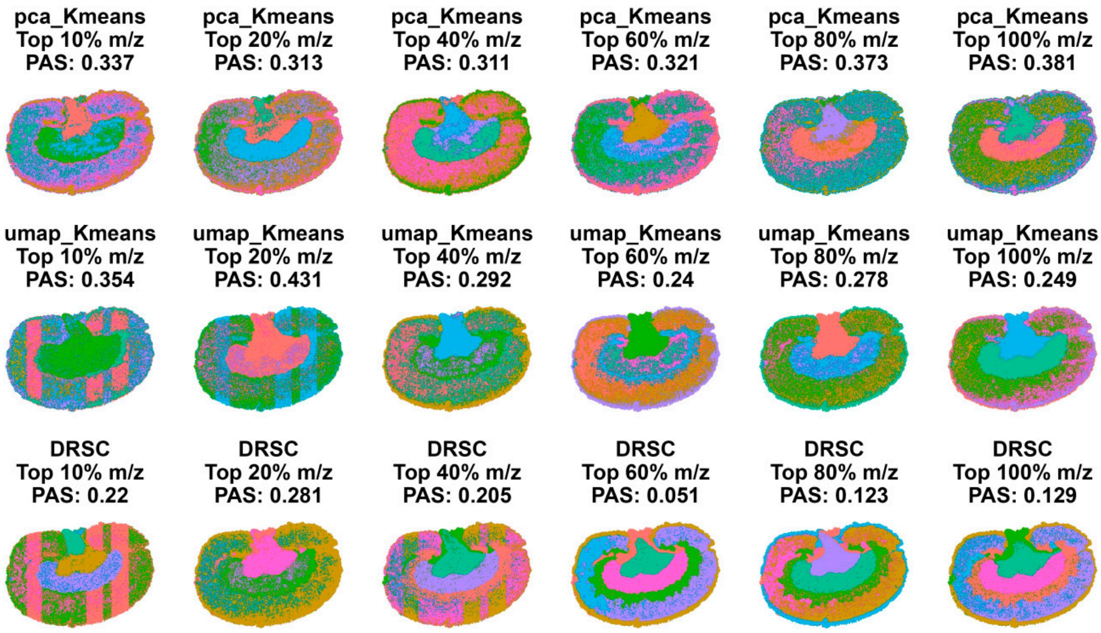

K. mfetus_neg

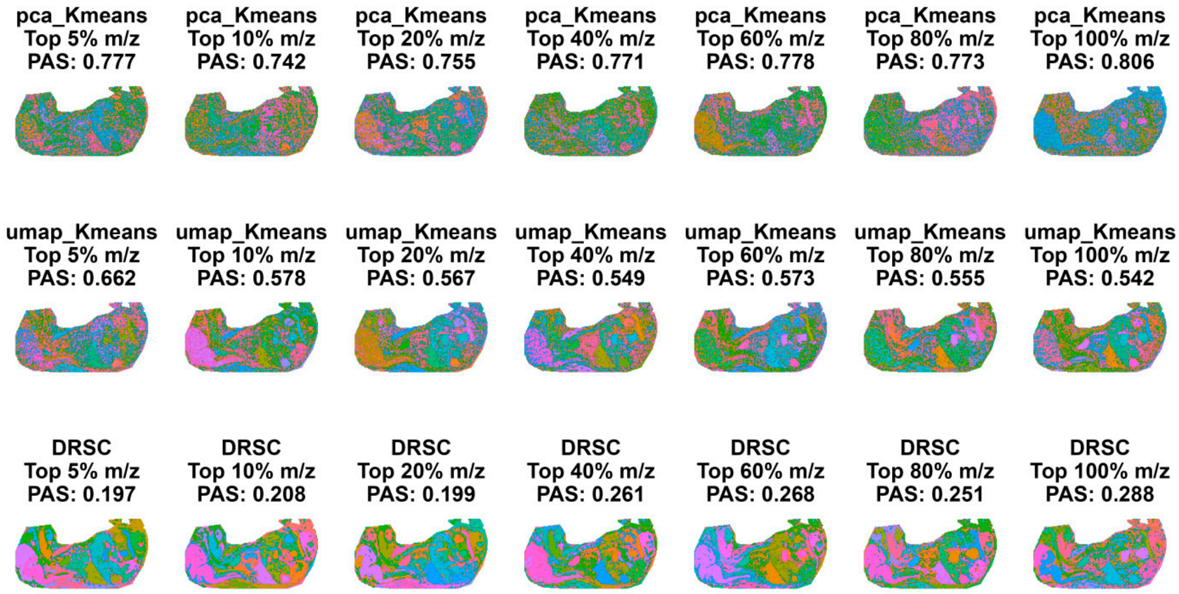

L.mbrain_neg40

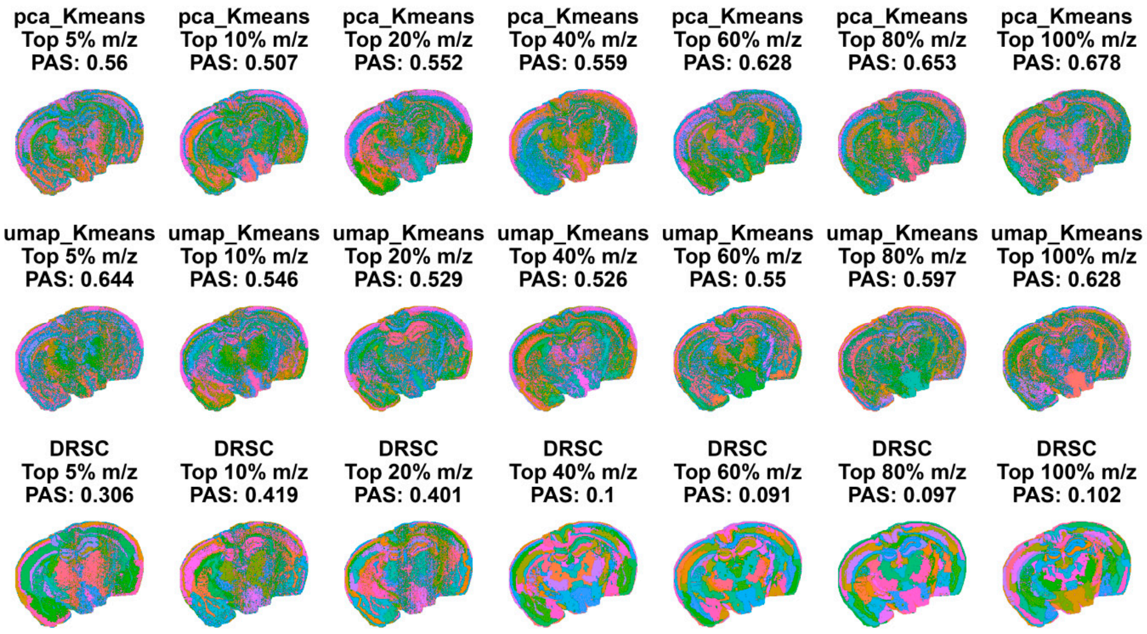

Figure S3. Spatial Clustering Maps Generated by All Evaluated Methods.

A. mbrain1_neg20

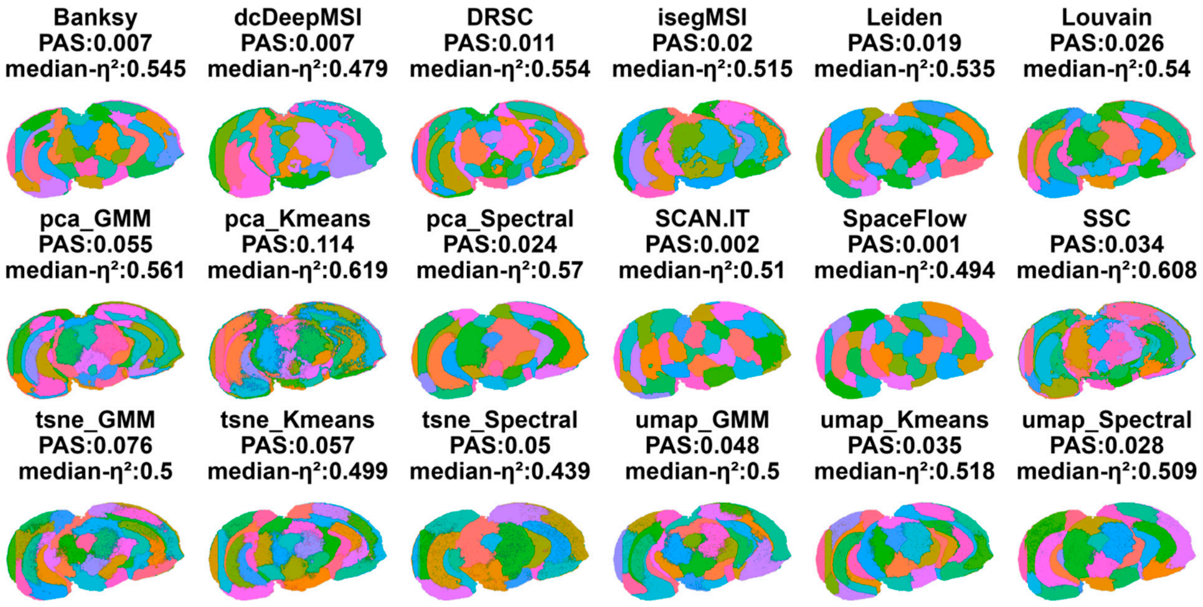

B. mbrain1_neg50

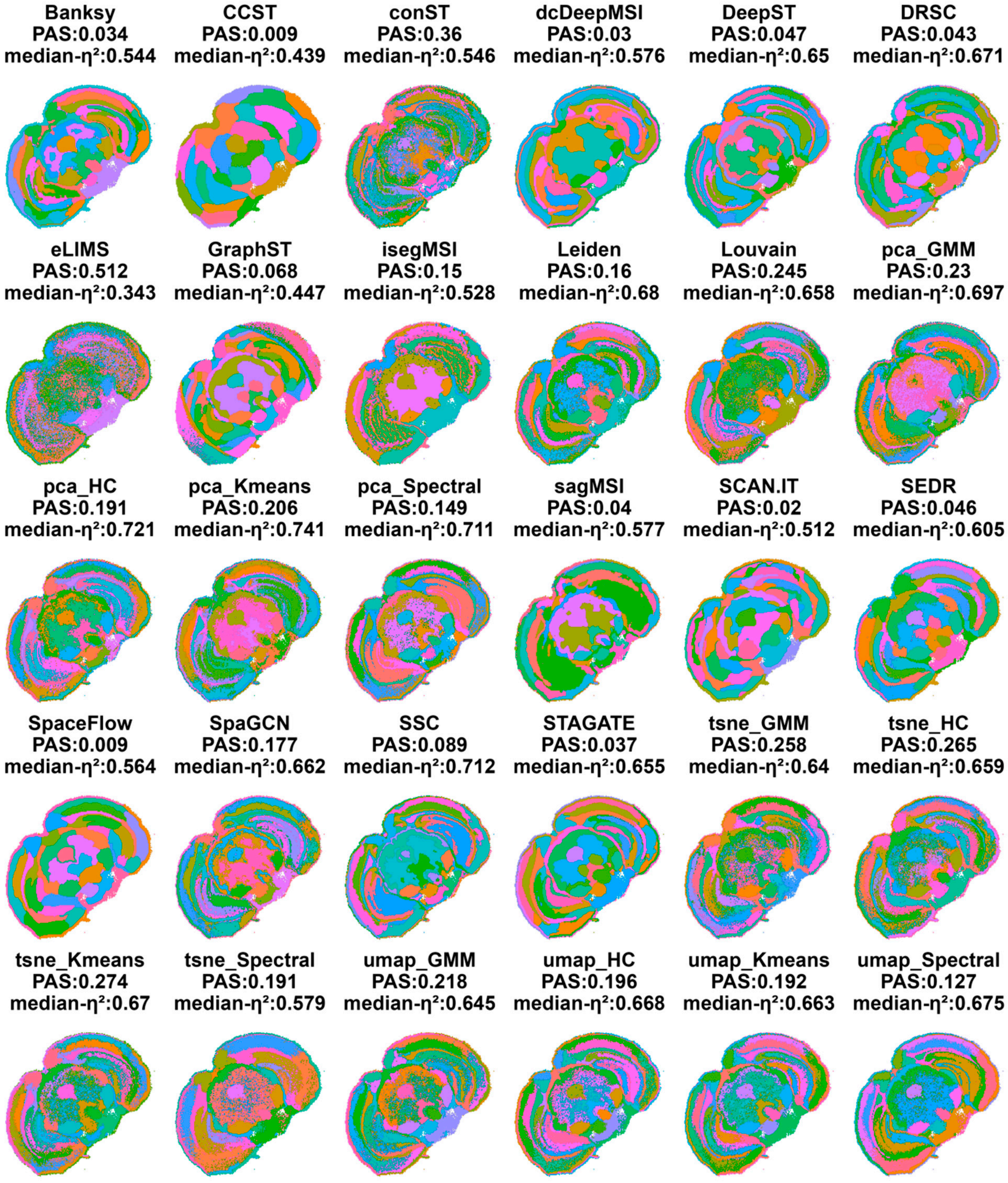

C. mbrain1_neg100

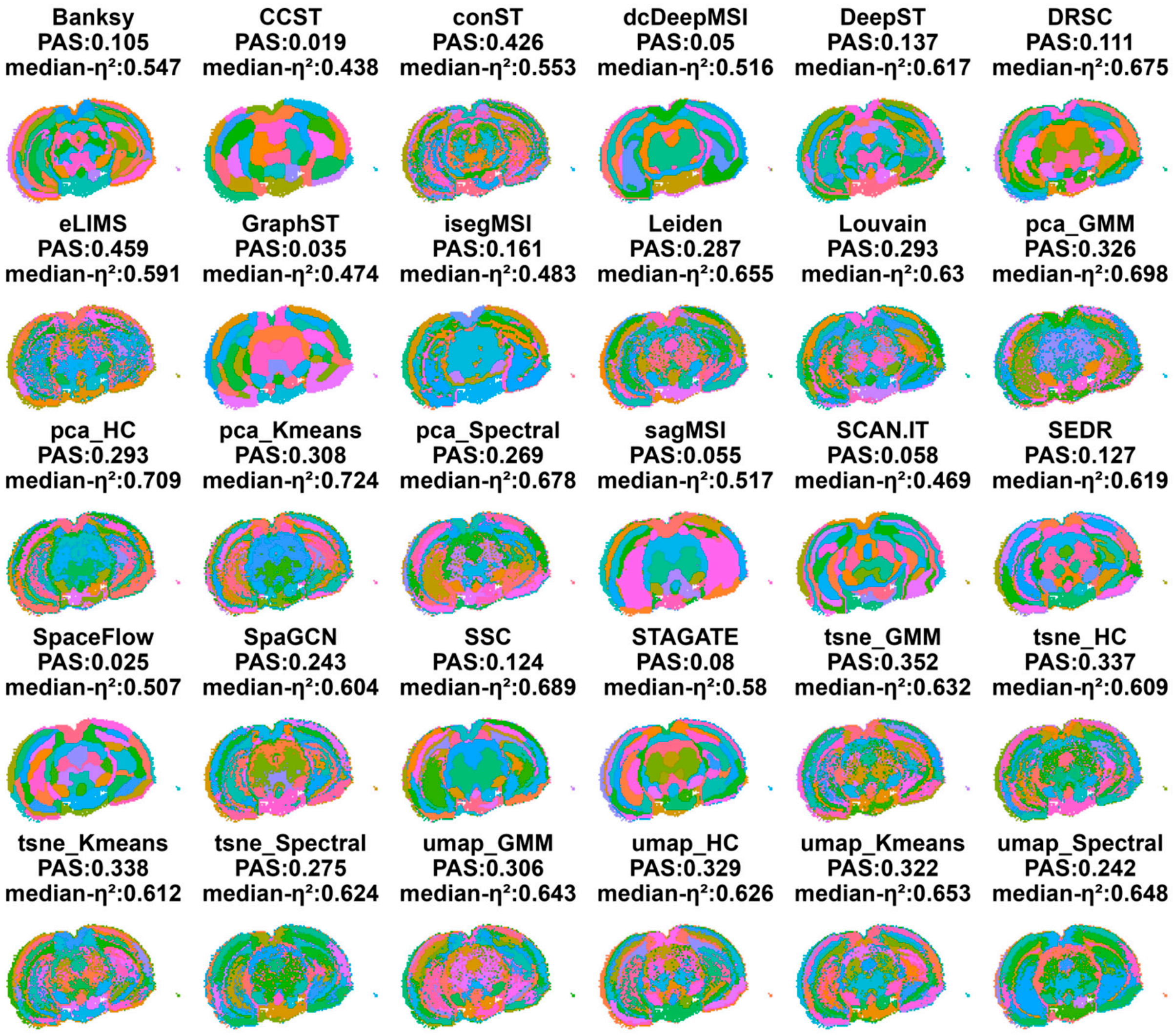

D. mbrain1_pos20

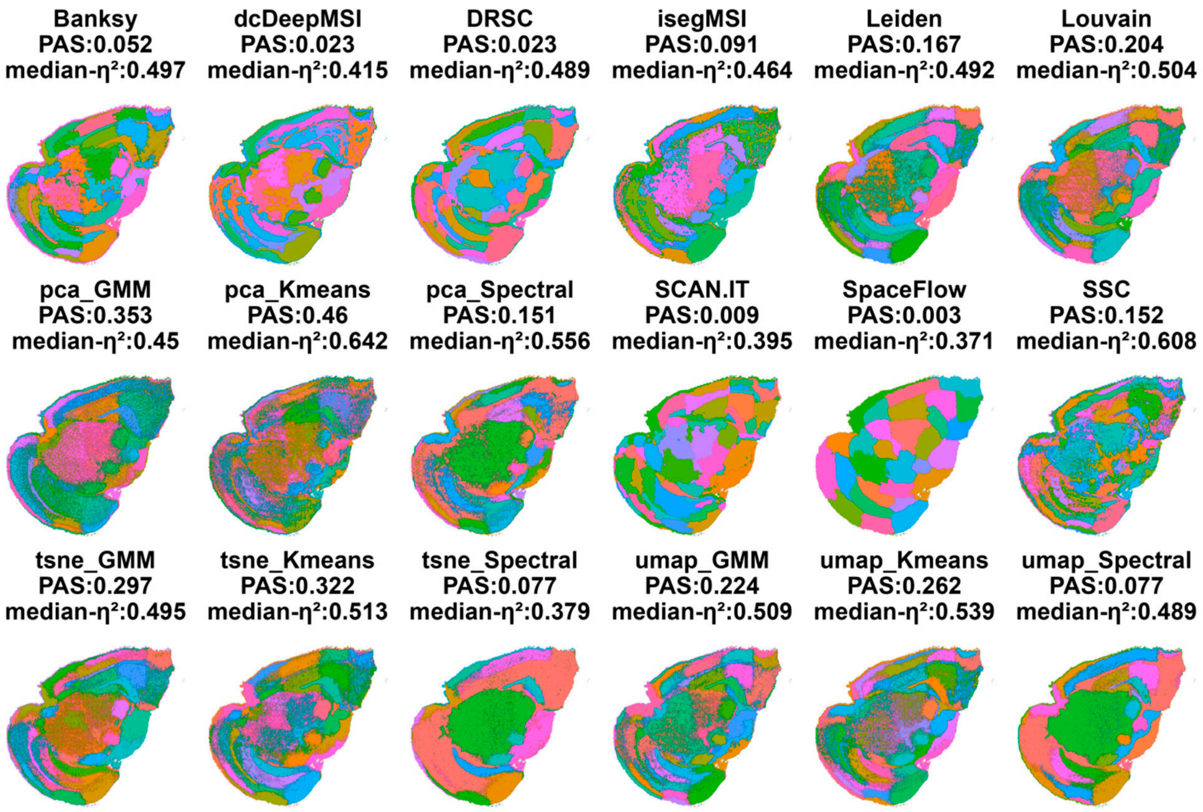

E. mbrain1_pos50

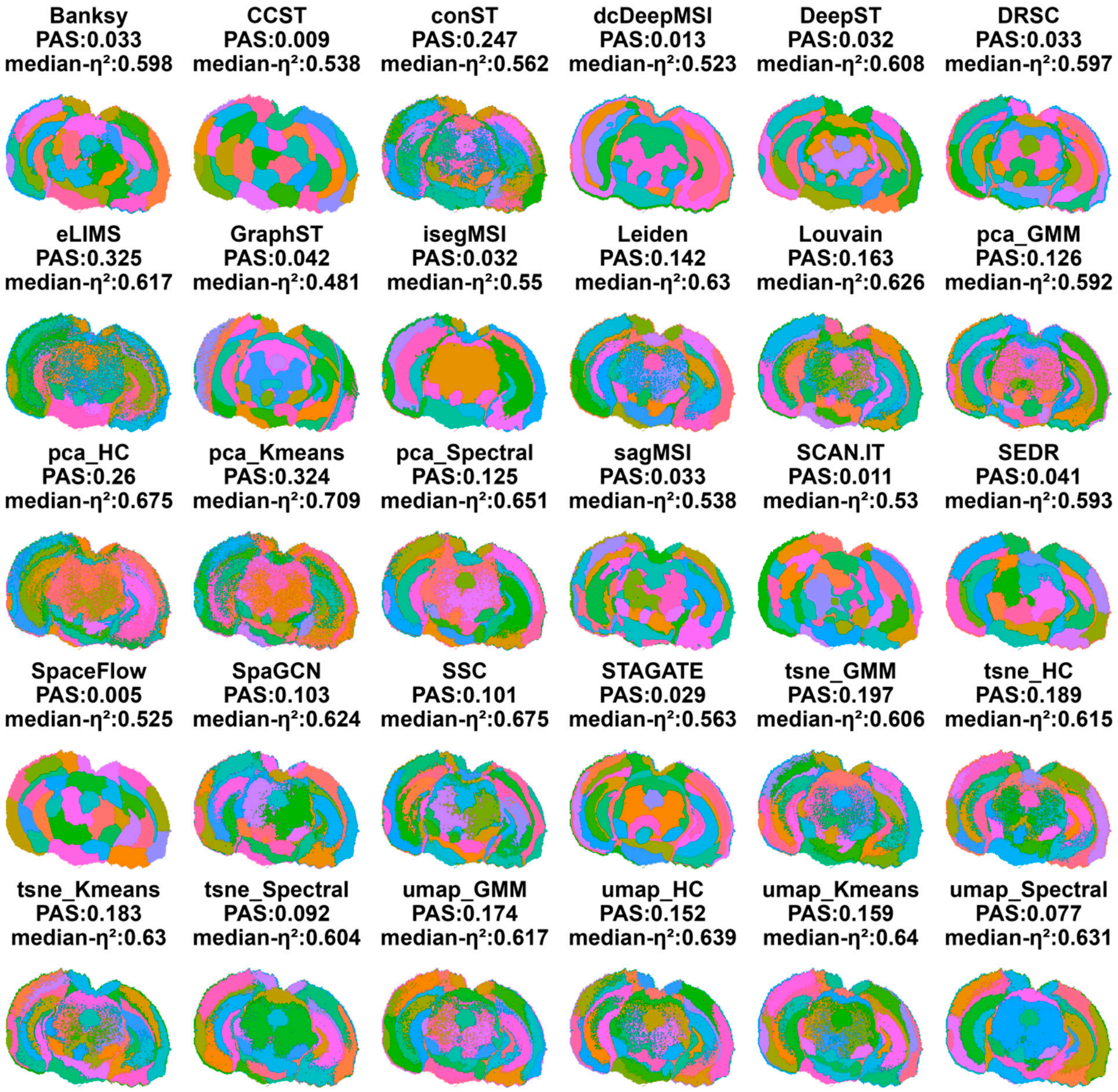

F. mbrain1_pos100

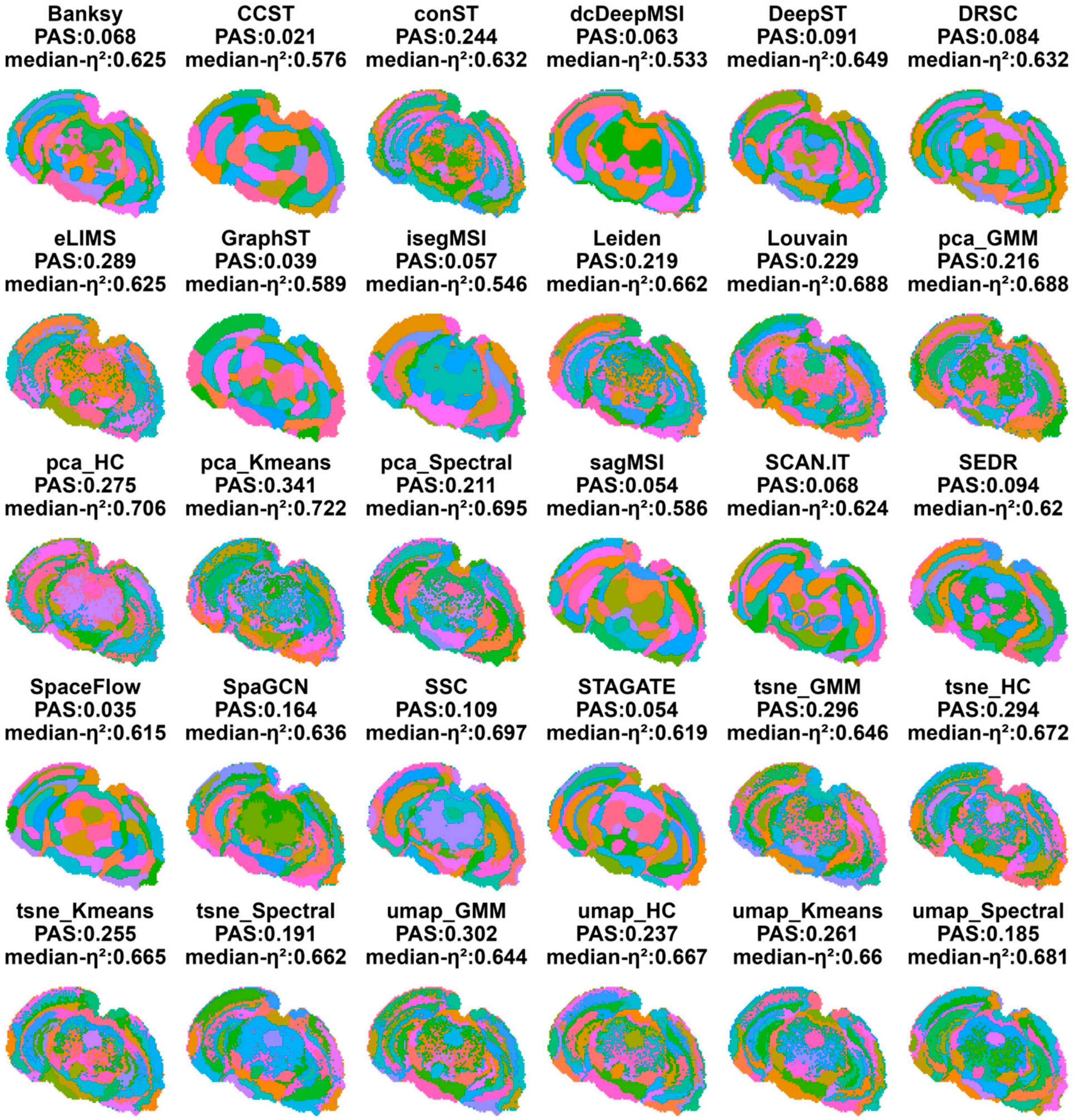

G. mbrain2_pos50

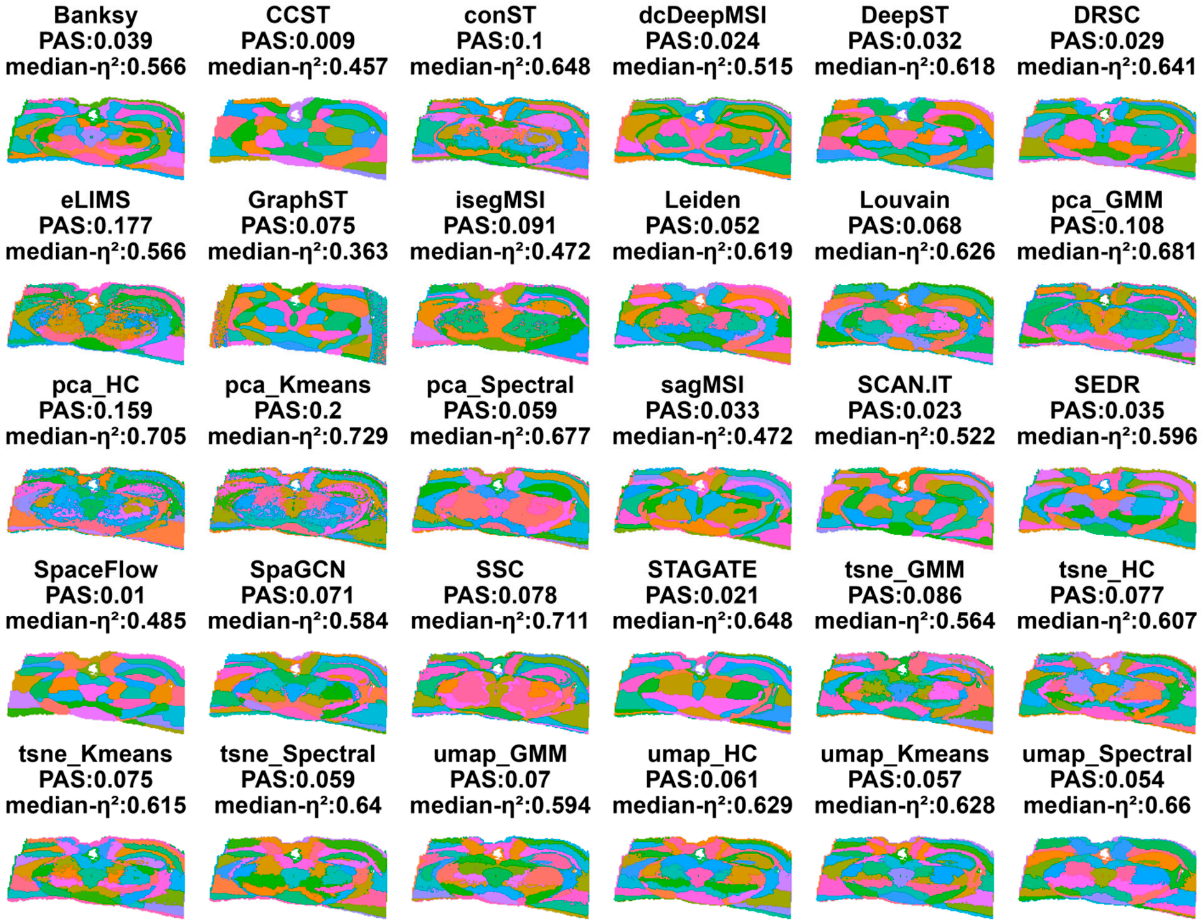

H. pfetus_neg

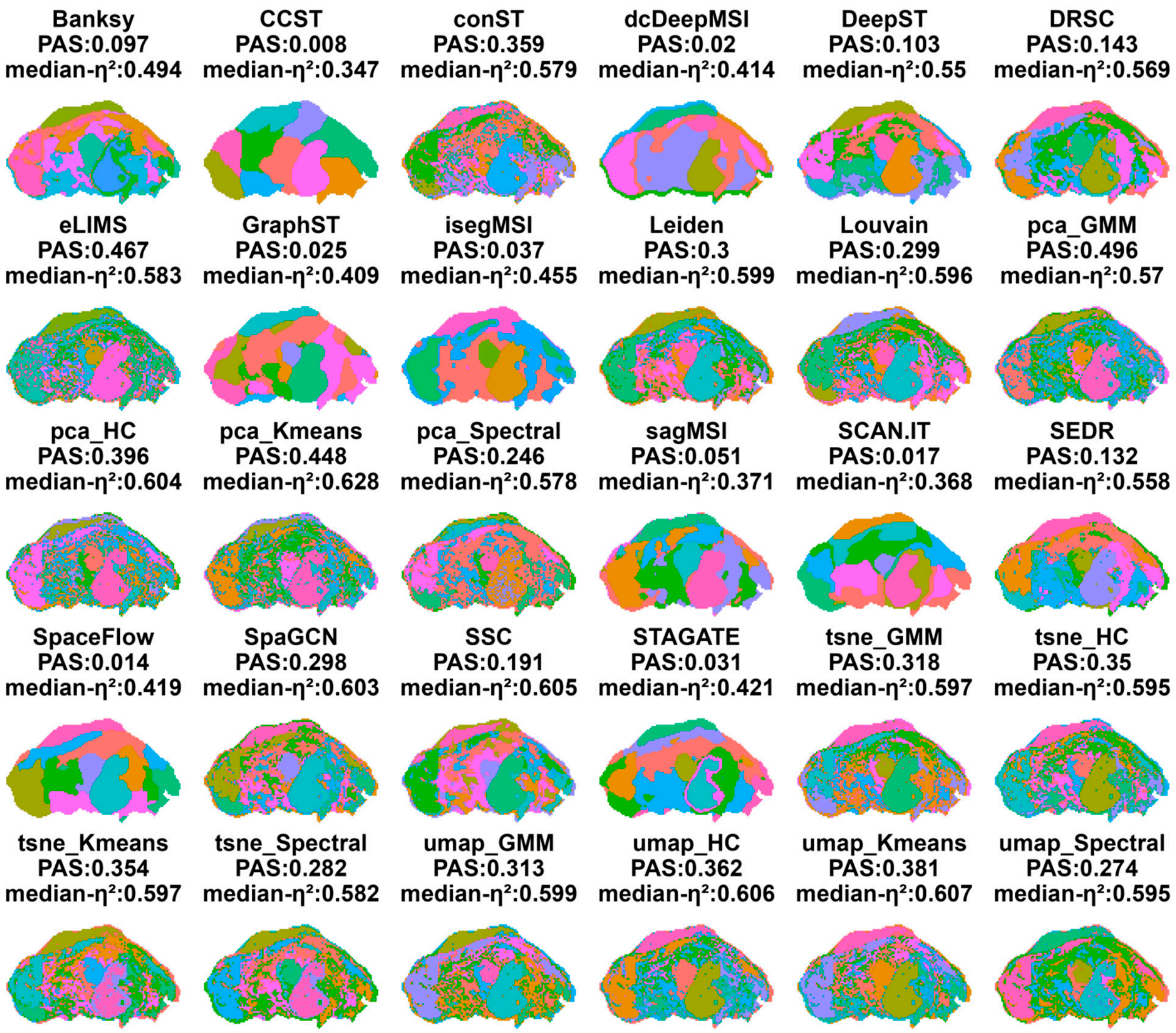
Banksy PAS:0.097 median-η^2:0.494
CCST PAS:0.008 median-η^2:0.347
conST PAS:0.359 median-η^2:0.579
dcDeepMSI PAS:0.02 median-η^2:0.414
DeepST PAS:0.103 median-η^2:0.55
DRSC PAS:0.143 median-η^2:0.569
eLIMS PAS:0.467 median-η^2:0.583
GraphST PAS:0.025 median-η^2:0.409
isegMSI PAS:0.037 median-η^2:0.455
Leiden PAS:0.3 median-η^2:0.599
Louvain PAS:0.299 median-η^2:0.596
pca_GMM PAS:0.496 median-η^2:0.57
pca_HC PAS:0.396 median-η^2:0.604
pca_Kmeans PAS:0.448 median-η^2:0.628
pca_Spectral PAS:0.246 median-η^2:0.578
sagMSI PAS:0.051 median-η^2:0.371
SCAN.IT PAS:0.017 median-η^2:0.368
SEDR PAS:0.132 median-η^2:0.558
SpaceFlow PAS:0.014 median-η^2:0.419
SpaGCN PAS:0.298 median-η^2:0.603
SSC PAS:0.191 median-η^2:0.605
STAGATE PAS:0.031 median-η^2:0.421
tsne_GMM PAS:0.318 median-η^2:0.597
tsne_HC PAS:0.35 median-η^2:0.595
tsne_Kmeans PAS:0.354 median-η^2:0.597
tsne_Spectral PAS:0.282 median-η^2:0.582
umap_GMM PAS:0.313 median-η^2:0.599
umap_HC PAS:0.362 median-η^2:0.606
umap_Kmeans PAS:0.381 median-η^2:0.607
umap_Spectral PAS:0.274 median-η^2:0.595

I. PDX_mbrain_pos100

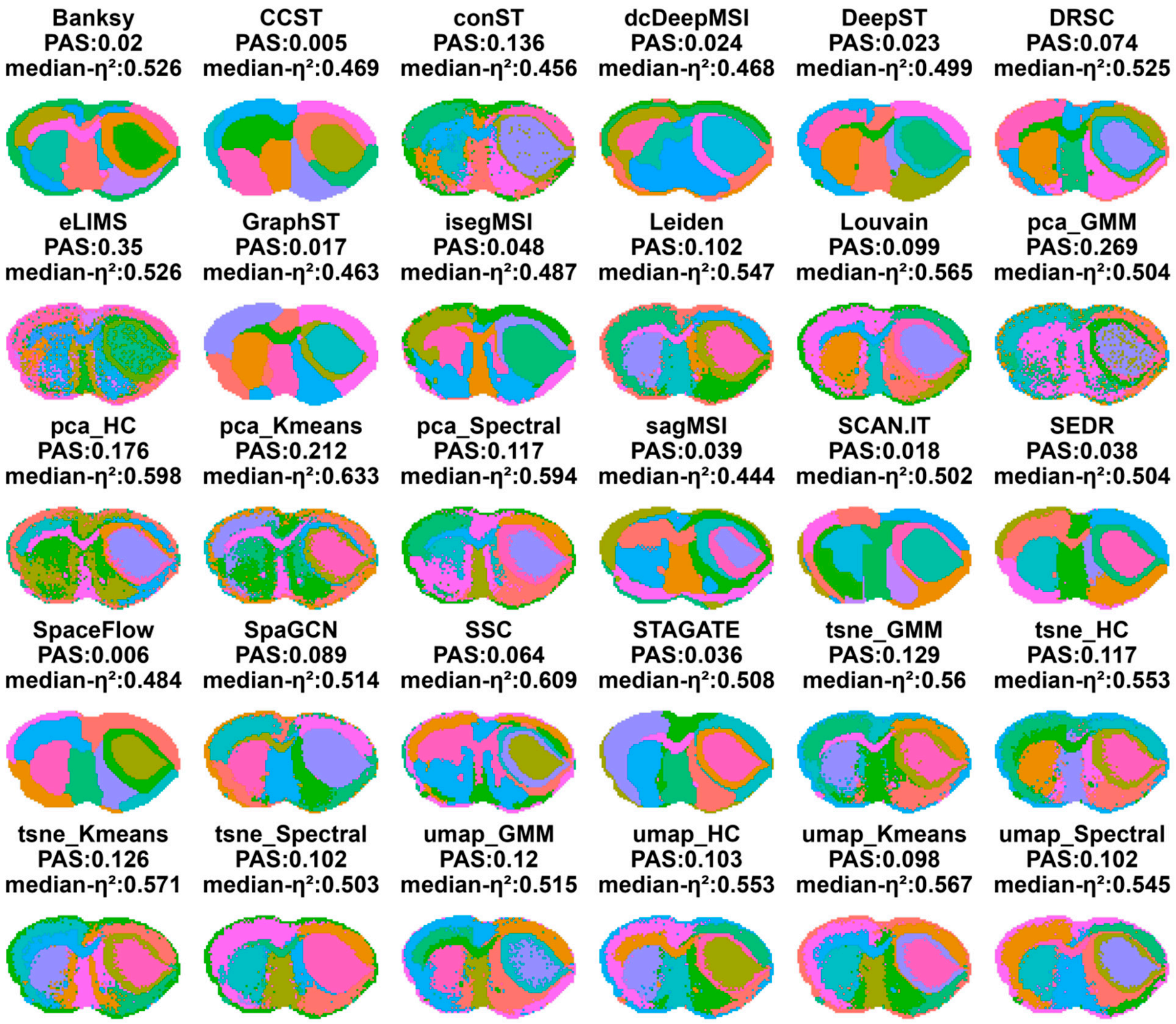

J. mkidney_neg40

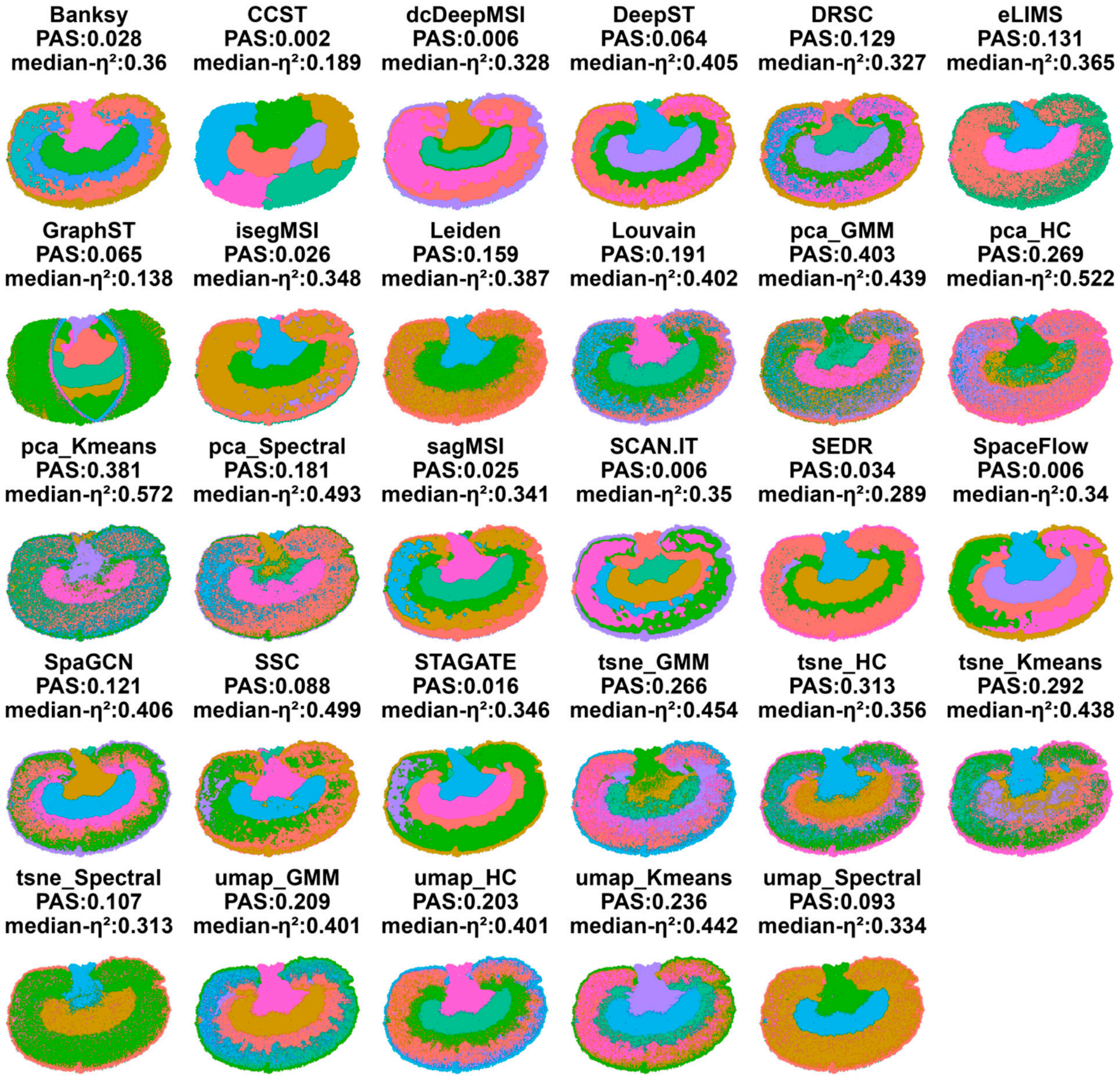

K. mfetus_neg

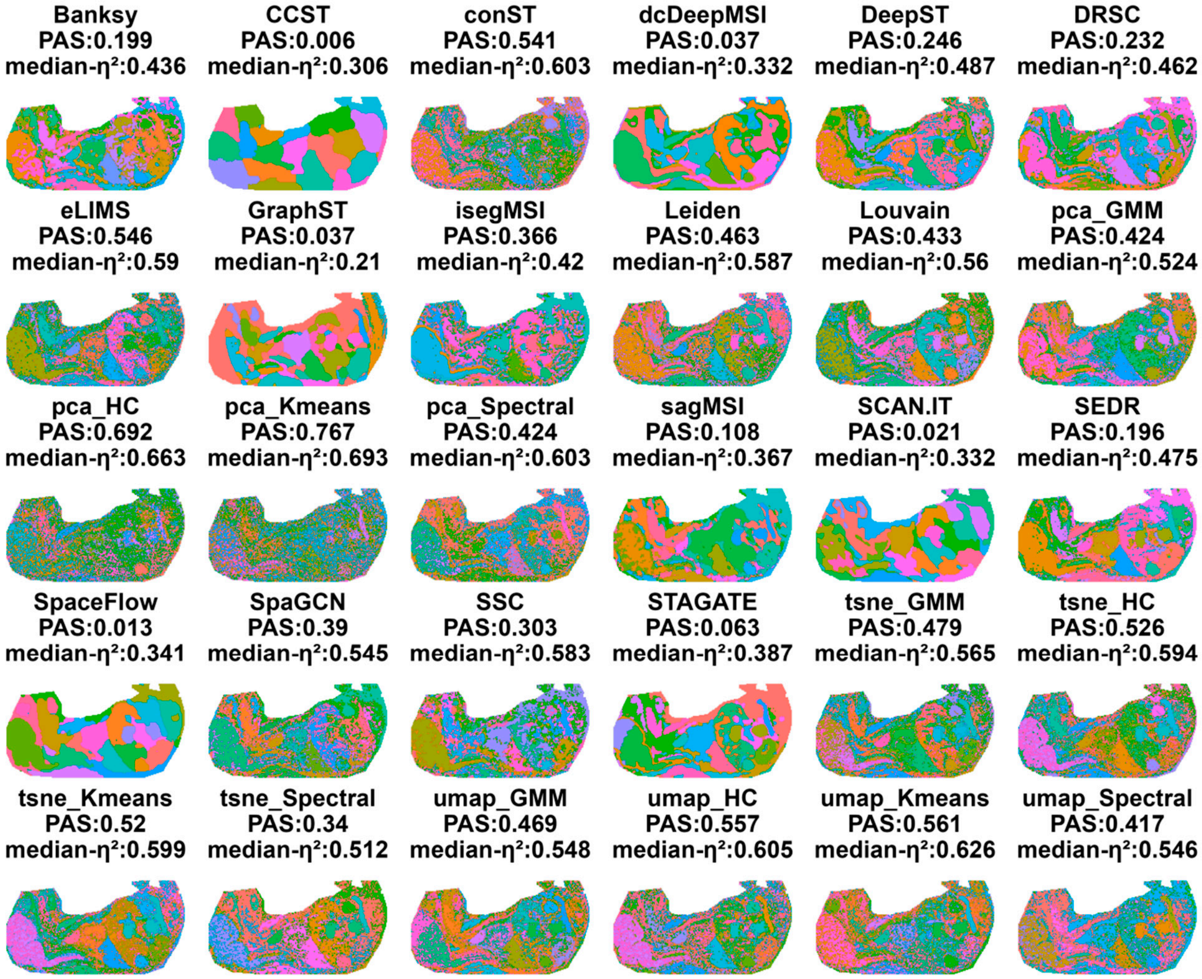

Banksy
PAS:0.199
median-η^2:0.436
CCST
PAS:0.006
median-η^2:0.306
conST
PAS:0.541
median-η^2:0.603
dcDeepMSI
PAS:0.037
median-η^2:0.332
DeepST
PAS:0.246
median-η^2:0.487
DRSC
PAS:0.232
median-η^2:0.462
eLIMS
PAS:0.546
median-η^2:0.59
GraphST
PAS:0.037
median-η^2:0.21
isegMSI
PAS:0.366
median-η^2:0.42
Leiden
PAS:0.463
median-η^2:0.587
Louvain
PAS:0.433
median-η^2:0.56
pca_GMM
PAS:0.424
median-η^2:0.524
pca_HC
PAS:0.692
median-η^2:0.663
pca_Kmeans
PAS:0.767
median-η^2:0.693
pca_Spectral
PAS:0.424
median-η^2:0.603
sagMSI
PAS:0.108
median-η^2:0.367
SCAN.IT
PAS:0.021
median-η^2:0.332
SEDR
PAS:0.196
median-η^2:0.475
SpaceFlow
PAS:0.013
median-η^2:0.341
SpaGCN
PAS:0.39
median-η^2:0.545
SSC
PAS:0.303
median-η^2:0.583
STAGATE
PAS:0.063
median-η^2:0.387
tsne_GMM
PAS:0.479
median-η^2:0.565
tsne_HC
PAS:0.526
median-η^2:0.594
tsne_Kmeans
PAS:0.52
median-η^2:0.599
tsne_Spectral
PAS:0.34
median-η^2:0.512
umap_GMM
PAS:0.469
median-η^2:0.548
umap_HC
PAS:0.557
median-η^2:0.605
umap_Kmeans
PAS:0.561
median-η^2:0.626
umap_Spectral
PAS:0.417
median-η^2:0.546

L. mbrain_neg40

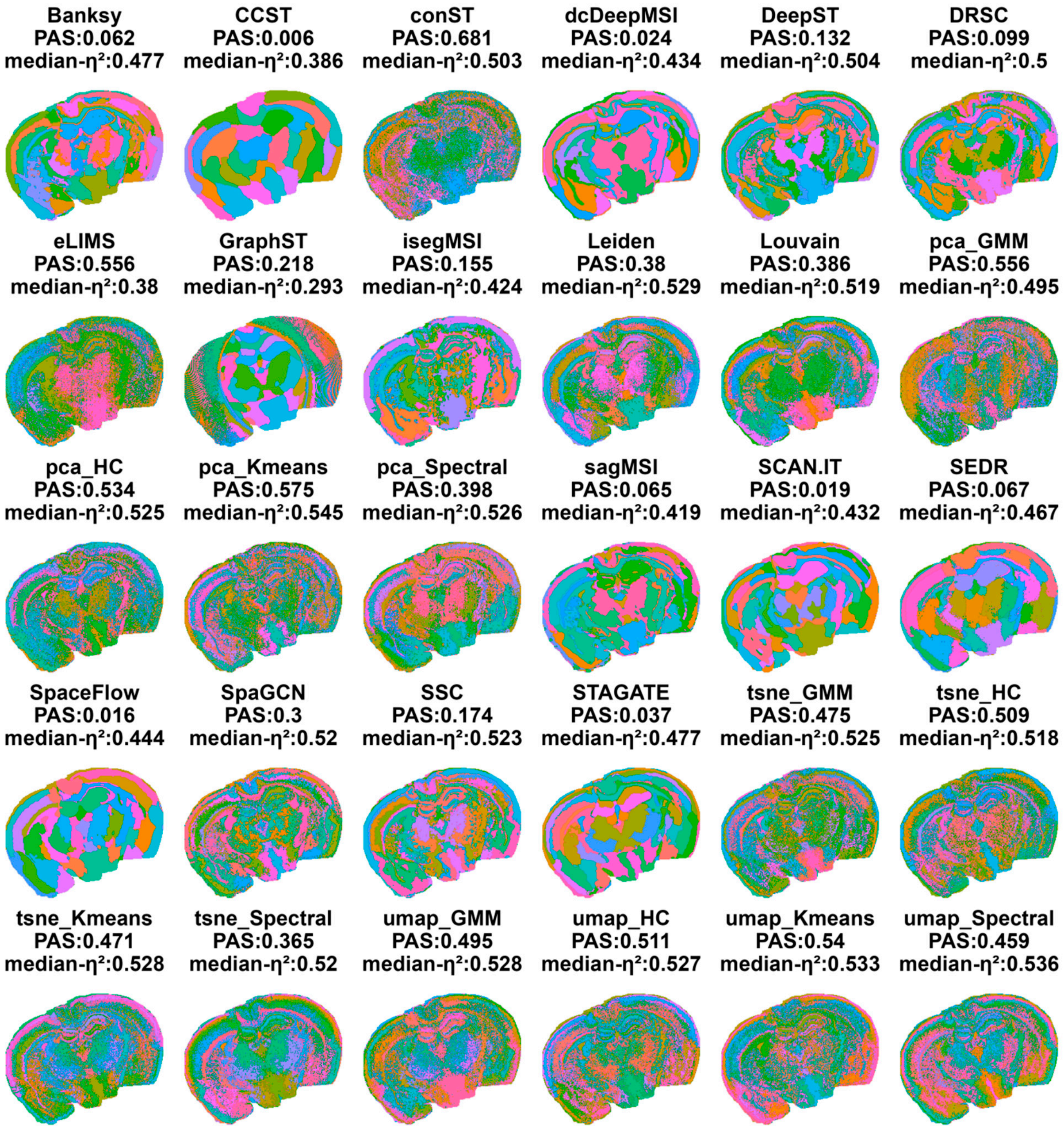

Figure S4. Validation of PAS and median-η² Using Alternative Spatial Continuity and Cluster-Separation Metrics.

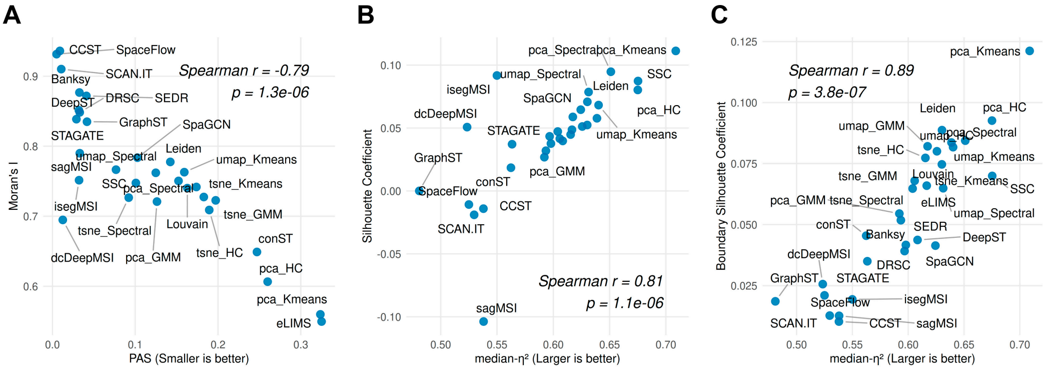

Figure S5. Joint Distributions of PAS and median-η² for All Methods Across Datasets.

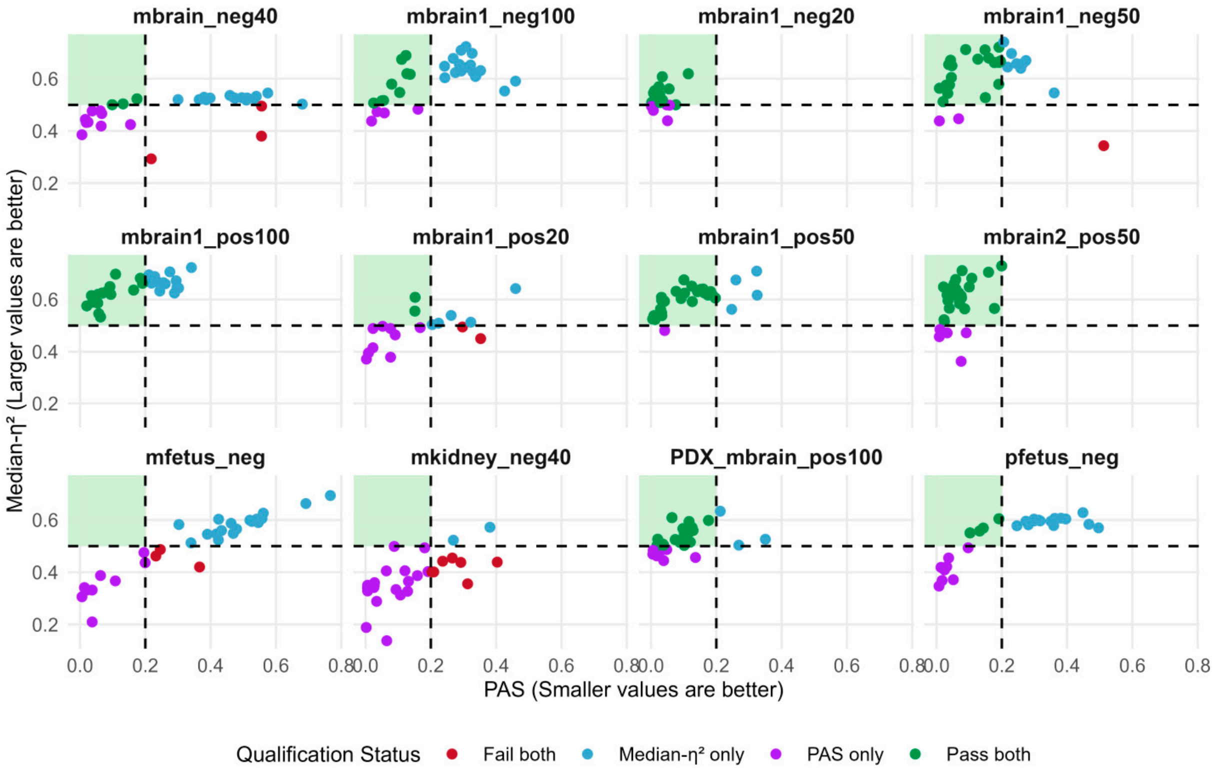

Figure S6. Pass Rates of Each Method under Dual Criteria of PAS and median-η^2 Across Datasets.

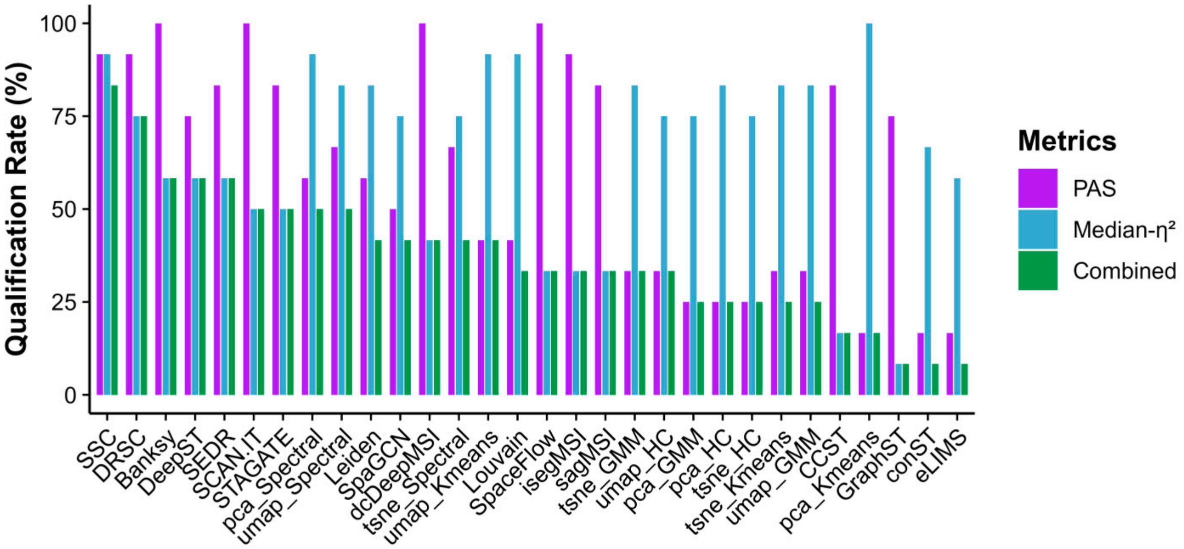

Figure S7. Pairwise Normalized Mutual Information (NMI) Between Clustering Results of Different Methods.

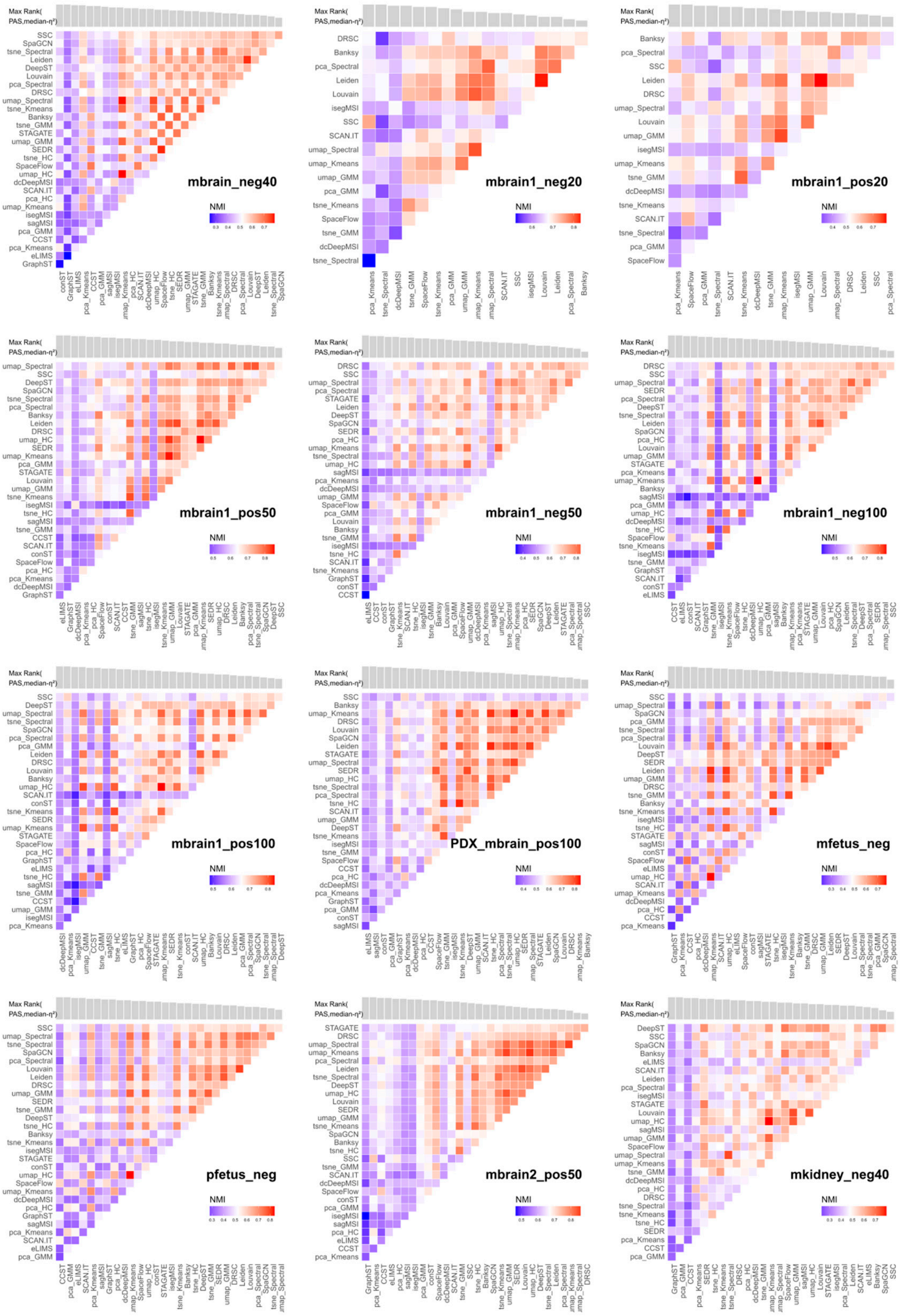
Max Rank(
PAS,median-η²)
NMI
mbrain_neg40
mbrain1_neg20
mbrain1_pos20
mbrain1_pos50
mbrain1_neg50
mbrain1_neg100
mbrain1_pos100
PDX_mbrain_pos100
mfetus_neg
pfetus_neg
mbrain2_pos50
mkidney_neg40

Figure S8. Distributions of Spatial Noise Score (SNS) in Filtered Datasets Used for Clustering.

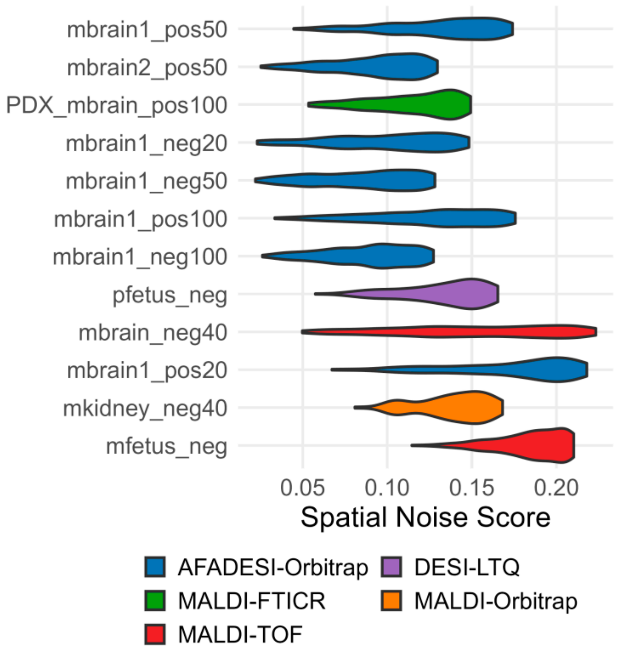

Figure S9. Robustness of Dual-Metric Evaluation under Different Threshold Stringencies.

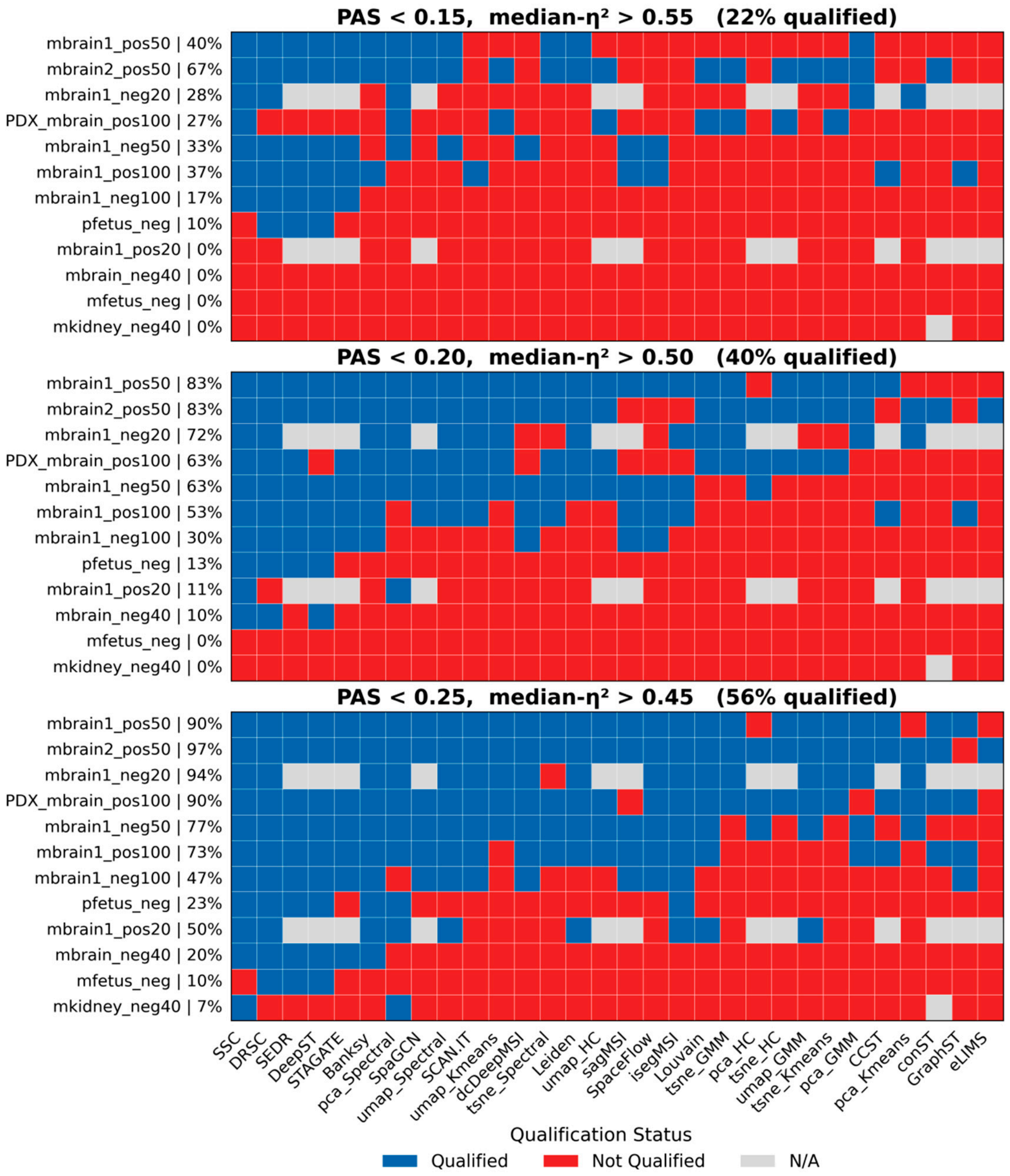

Figure S10. Computational Efficiency of Clustering Methods Across Datasets.

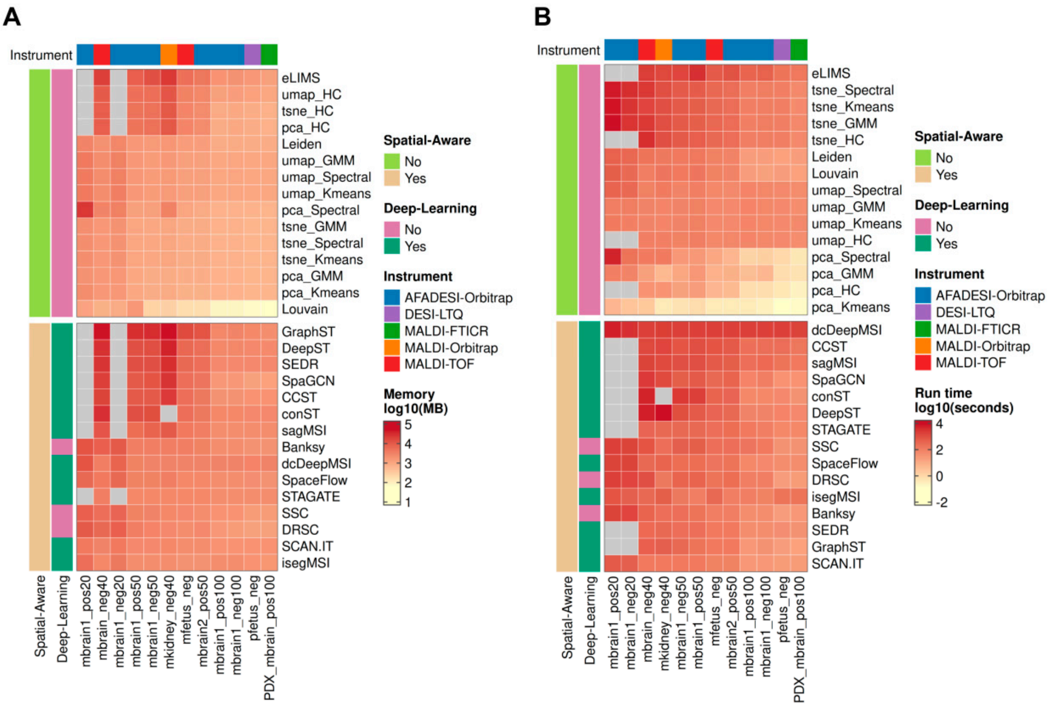

Figure S11. Workflow Reproduction and Benchmarking Results on the SMcluster Online Platform.

A. User interface for data import and preview.

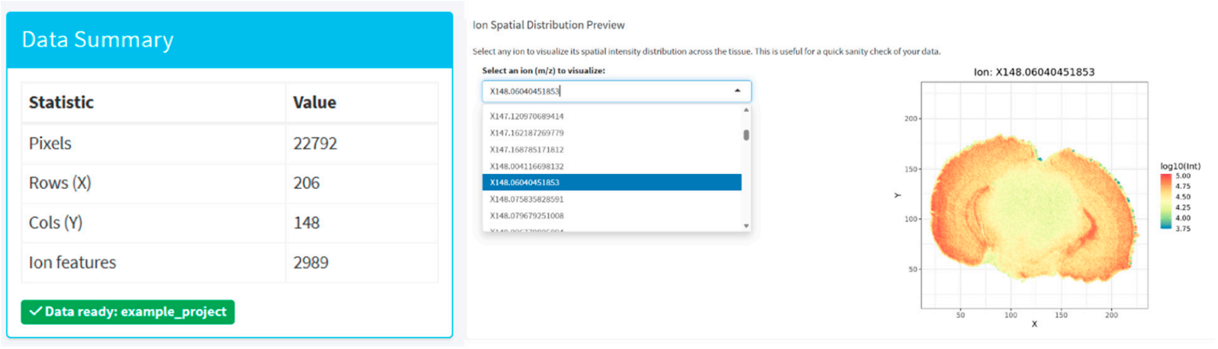

B. Interactive interface for ion filtering based on Spatial Noise Score (SNS) thresholds.

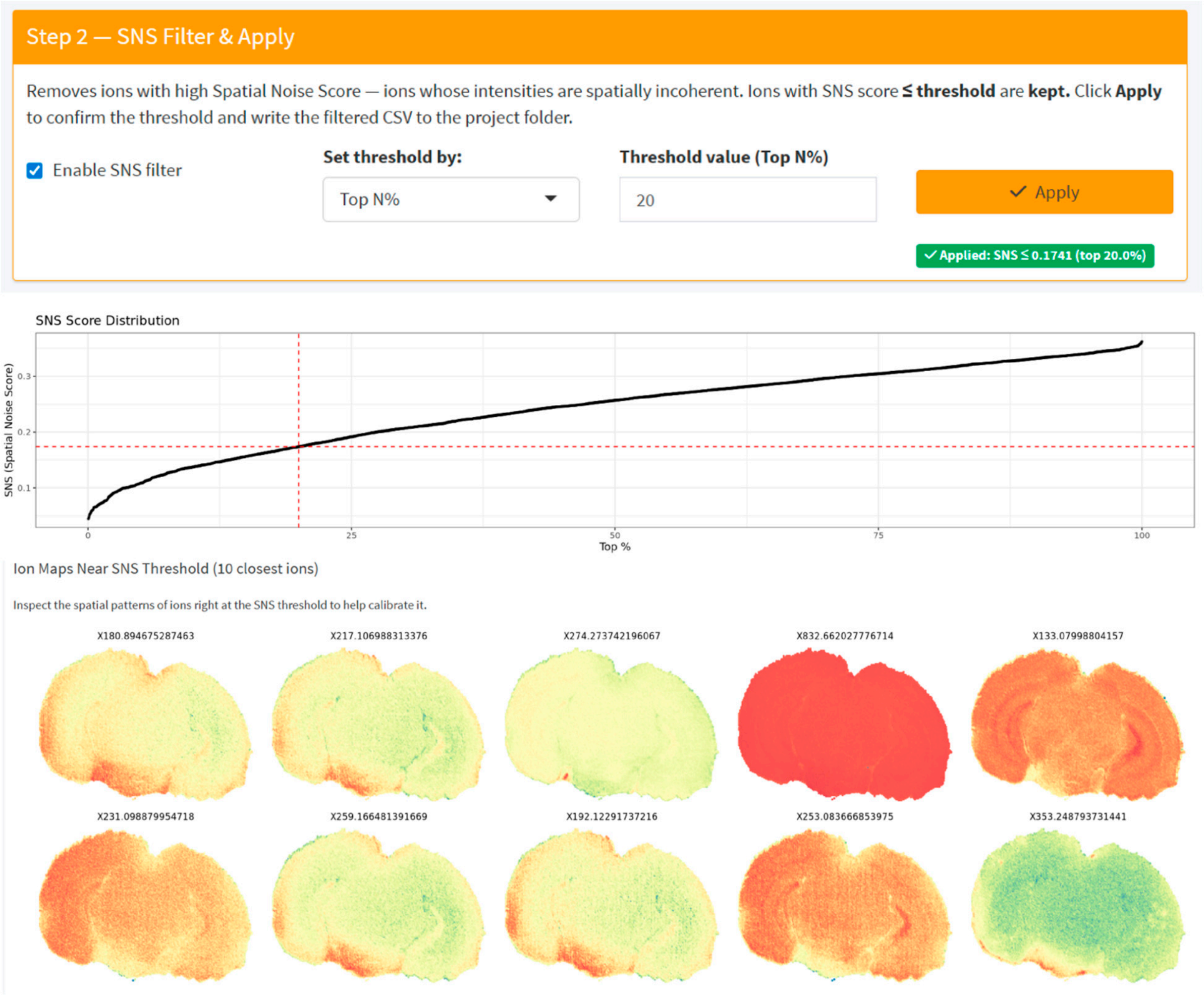

C. Configuration panel for selecting and parameterizing clustering algorithms.

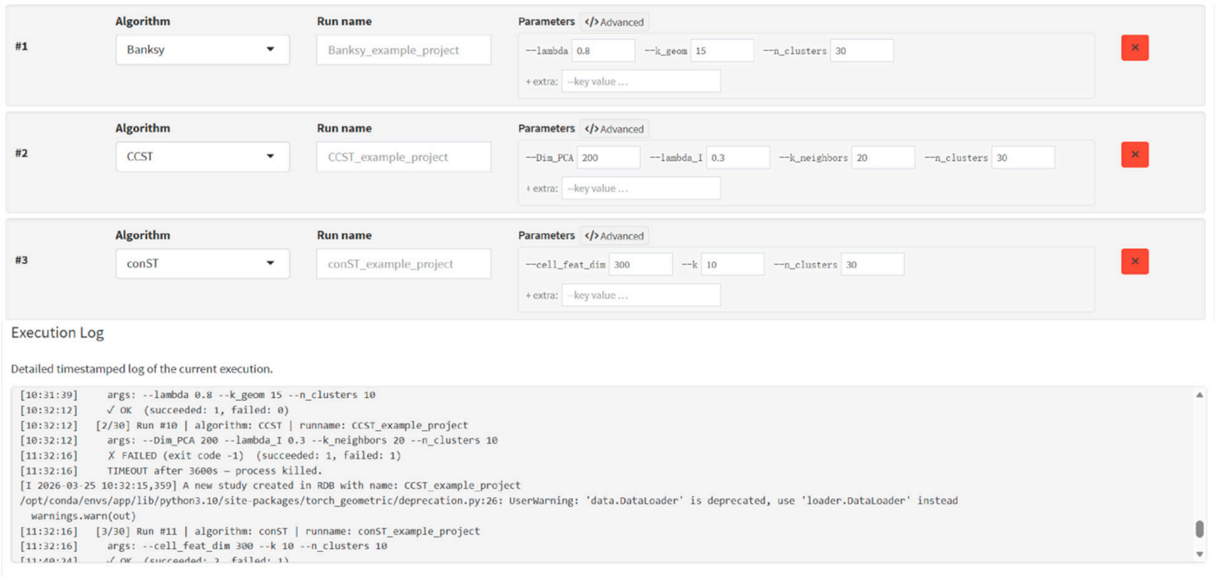

D. Dashboard for visualizing clustering results and quantitative evaluation metrics.

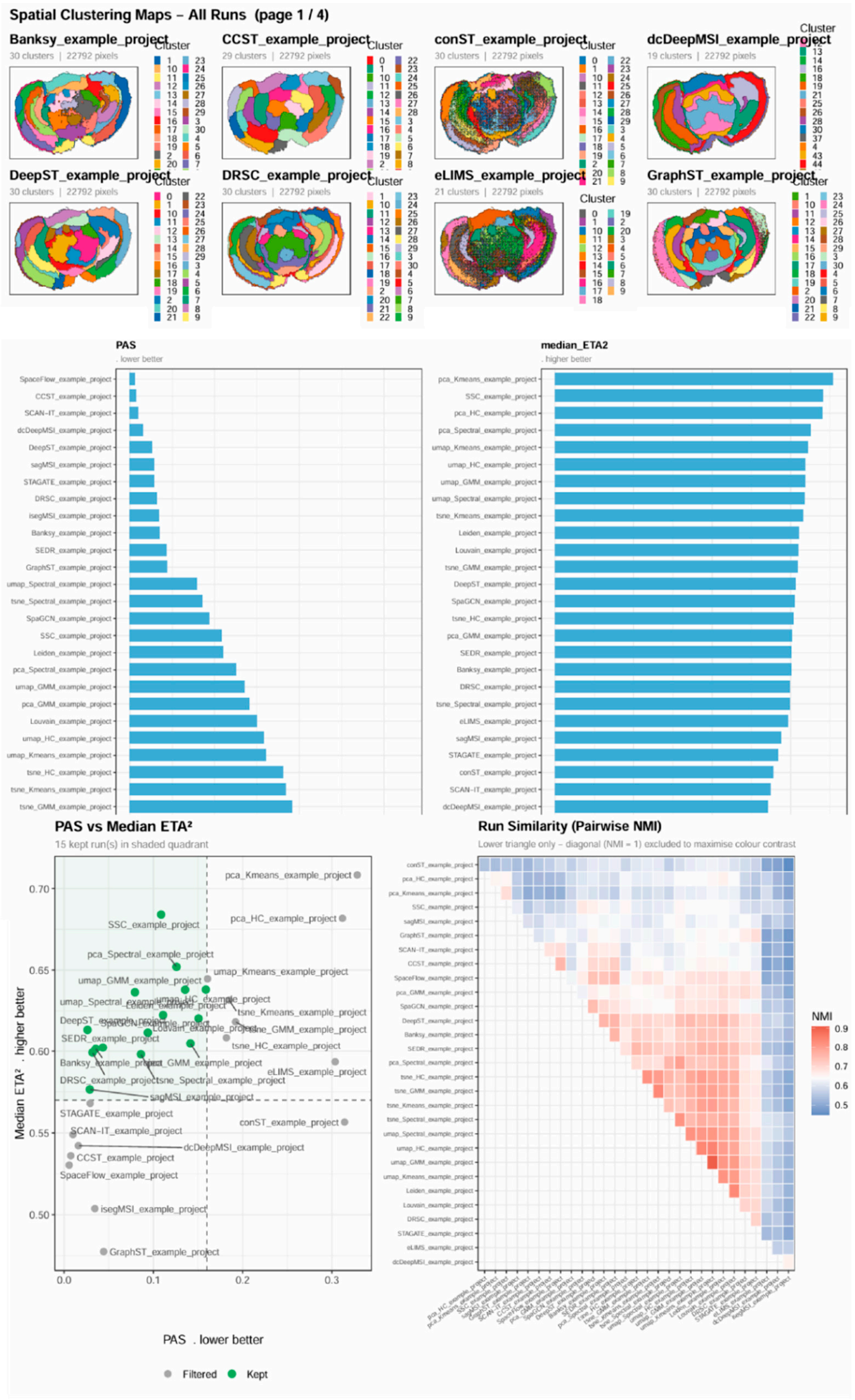

Figure S12. Evaluation of an Independent Mouse Uterine MSI Dataset.

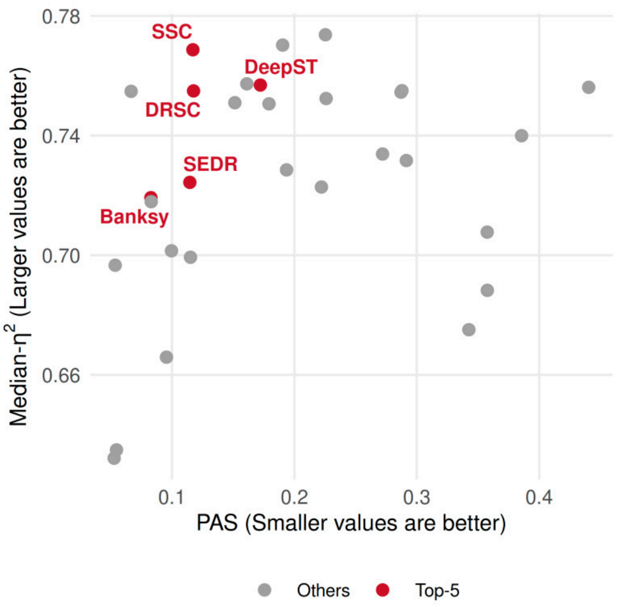

Figure S13. Comparison of Clustering Results under Different SNS Filtering Stringencies (Retaining Top 20% vs. Top 80% m/z).

A. PAS comparison between filtering thresholds: top 20% vs. top 80%

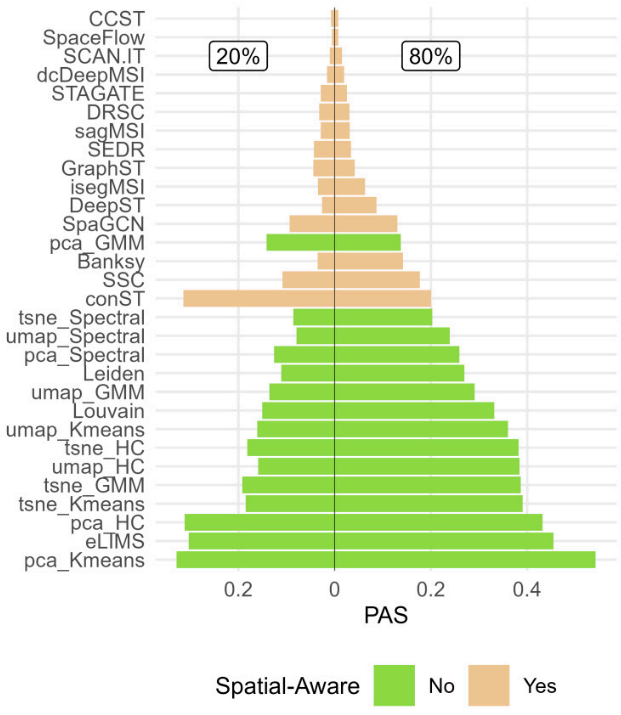

B. Spatial clustering map using ions with the top 20% m/z

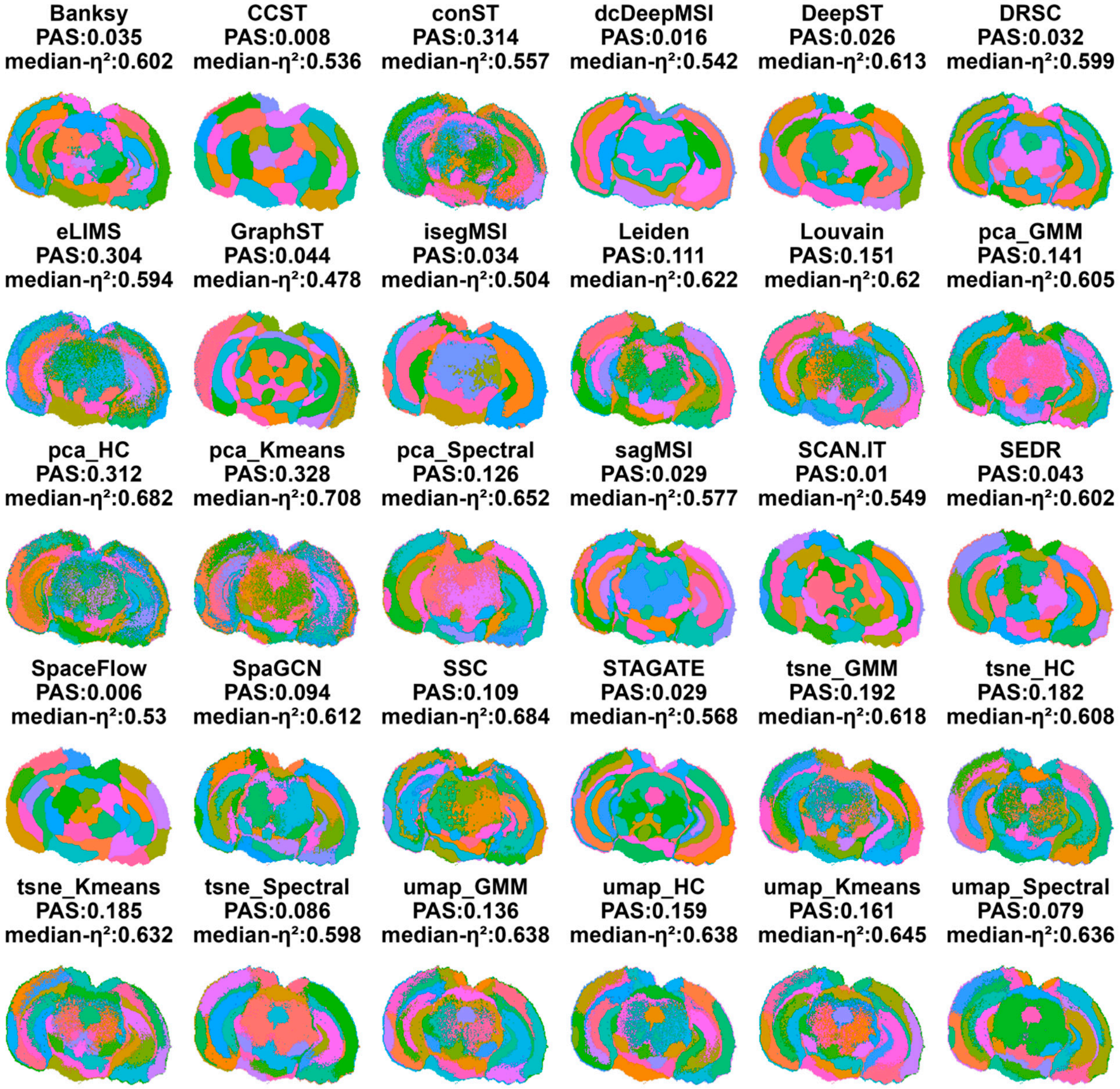

C. Spatial clustering map using ions with the top 80% m/z

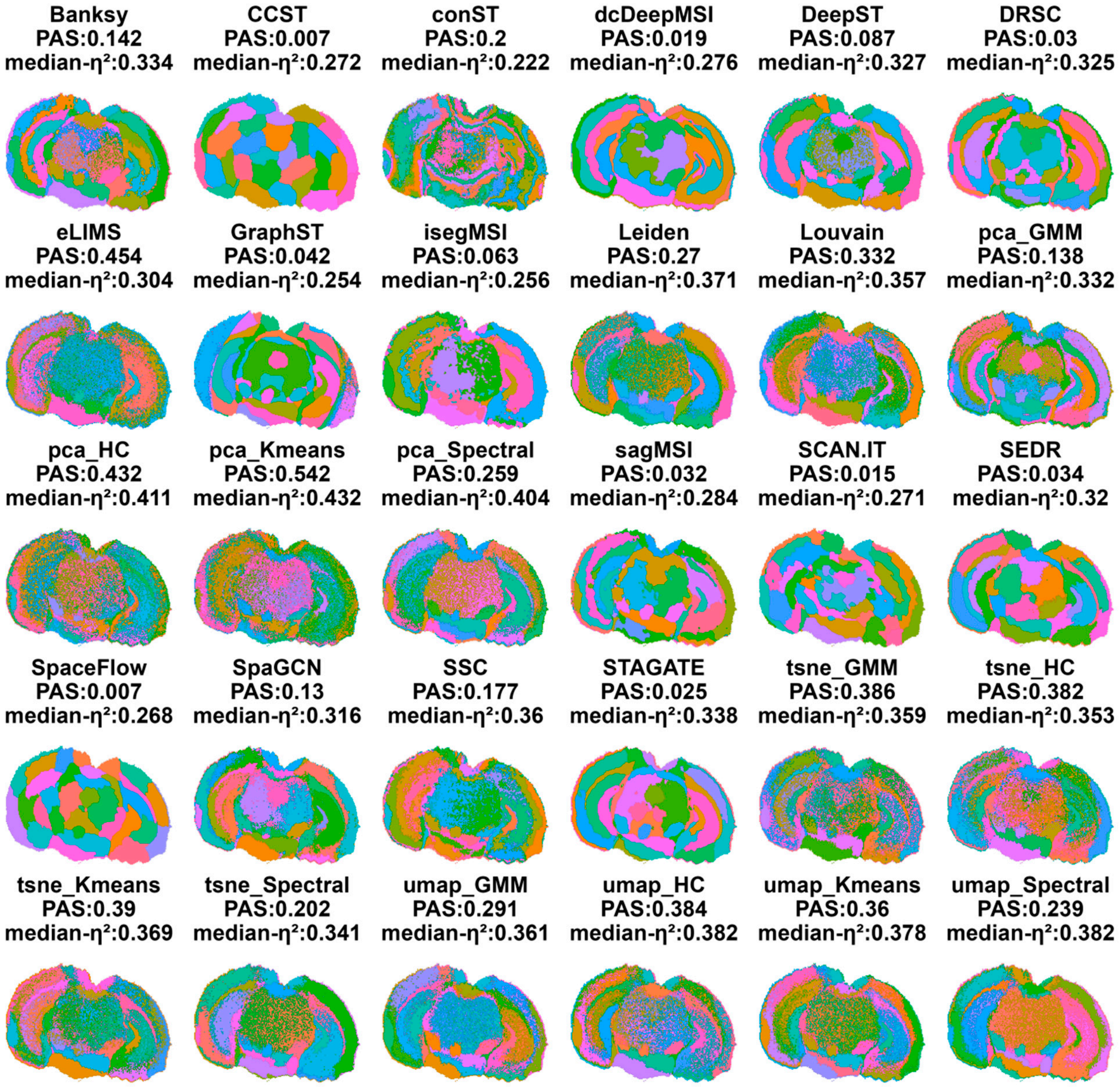

Supplement: Supplementary file 1 [file metabolites-16-00348-s001.zip › metabolites-4290184-supplementary.pdf]
